# The unified protocol as an internet-based intervention for emotional disorders: Randomized controlled trial

**DOI:** 10.1371/journal.pone.0270178

**Published:** 2022-07-11

**Authors:** Carmen Schaeuffele, Sophie Homeyer, Luis Perea, Lisa Scharf, Ava Schulz, Christine Knaevelsrud, Babette Renneberg, Johanna Boettcher

**Affiliations:** 1 Department of Education and Psychology, Freie Universität Berlin, Berlin, Germany; 2 Department of Psychiatry, Psychotherapy and Psychosomatics, University of Zurich, Zürich, Switzerland; 3 Clinical Psychology and Psychotherapy, Psychologische Hochschule Berlin, Berlin, Germany; UNITED STATES

## Abstract

The Unified Protocol (UP) as a transdiagnostic intervention has primarily been applied in the treatment of anxiety disorders and in face-to-face-settings. The current study investigated the efficacy of a 10-week Internet-based adaptation of the UP for anxiety, depressive, and somatic symptom disorders. The trial was registered under DRKS00014820 at the German Clinical Trial Registry, DRKS. Participants (*n* = 129) were randomized to treatment or waitlist control. Significant treatment effects were found for symptom distress, satisfaction with life, positive/negative affect and markers of anxiety, depression, and somatic symptom burden (within-group *Hedges’ g* = 0.32–1.38 and between-group *g* = 0.20–1.11). Treatment gains were maintained at 1- and 6-month-follow-up. Subgroup analyses showed comparable effects in participants with anxiety and depressive disorders. 26.6% dropped out of treatment and 35.38% did not provide post-treatment assessments. The results strengthen the application of the UP as an Internet-based treatment for alleviating symptom distress across emotional disorders. More research on the applicability for single disorders is needed and avenues to improve adherence and attrition rates should be explored.

## Introduction

About a third of the German population fulfilled criteria for at least one mental disorder in the previous 12 months [1], the so-called emotional disorders being amongst the most prevalent. Emotional disorders are an umbrella term for disorders that are characterized by shared mechanisms of onset and maintenance. Their core features include heightened emotionality, a negative reaction towards these emotions and efforts to decrease the emotion [2]. Following this definition, emotional disorders subsume disorders like anxiety and depressive disorders as well as related disorders like somatic symptom disorders. The emotional disorders do not only represent the most common diagnoses but they are also highly comorbid [1]. While comorbidity results in greater severity, impairment, and chronicity [3–5], the majority of evidence-based treatments is focused on treating single disorders [6]. Transdiagnostic treatments, that simultaneously target several disorders, address comorbidity and have been applied successfully in face-to-face [7, 8] and Internet-based settings [9, 10].

While there is a growing evidence base for transdiagnostic treatments, transdiagnostic treatments encompass heterogeneous approaches that vary considerably in their foundation in theory [11]. In addition, the majority of treatments are applied exclusively in Internet-based or face-to-face settings [7–10]. Although applying transdiagnostic treatments may improve access to evidence-based treatments [12], the growing number of new transdiagnostic treatments and the divide between both delivery formats may impede dissemination. Instead, it seems reasonable to concentrate on effective transdiagnostic treatments that explicitly target the underlying mechanisms shared by emotional disorders and are adaptable across different settings and populations, like the Unified Protocol (UP) [12–14]. The UP is a transdiagnostic cognitive behavioral treatment for emotional disorders, originally developed for the face-to-face setting [15]. In the UP’s understanding of emotional disorders it is not the frequency or intensity with which individuals experience negative emotions, but how they react to these negative emotions that characterizes these disorders [2, 16]. These dysfunctional reactions to emotions, e.g., negative appraisal or avoidance tendencies, constitute the main treatment targets in the UP. Following these considerations, the UP should not only be suitable to target anxiety disorders but also disorders like insomnia, eating or depressive disorders [2, 16]. This would also extend to somatic symptom disorders, for which anxiety and preoccupation about physical symptoms as well as avoidance and reassurance seeking are key diagnostic criteria [17]. Previous studies found that the UP led to moderate to large effect sizes compared to waitlist and showed equivalence to gold-standard single-disorder protocols for mixed anxiety disorders [18, 19]. The UP has been delivered over the Internet as a 10-week guided intervention in a first preliminary study for anxiety and depression [20]. Tulbure and colleagues (2018) found that an Internet-based adaptation of the UP led to medium to large effects for anxiety and depression. While this is encouraging of the UP’s potential as an Internet-based intervention, the study sample excluded participants with severe symptoms and showed a lower number of diagnoses in comparison to face-to-face applications. In addition, the trial suffered from high attrition.

So far, the majority of research efforts have been concentrated on the UP’s effectiveness for anxiety and the evidence base beyond anxiety disorders is still limited [13]. While a meta-analysis found that the UP led to medium to large effects across anxiety and depression [21], the trials that did investigate the UP for primary depression are single case studies and open trials [15, 22–26]. The lack of more rigorous study designs to examine depression may limit the validity of the findings on the UP’s effectiveness for depression. The third most prevalent group of emotional disorder are somatic symptom disorders [1]. Despite their prevalence in primary care, their high medical health care utilization as well as high risk of chronicity and disability [27], somatic symptom disorders have not been investigated within the UP framework.

Our goal for this study was to extend the evidence base for the UP in an Internet-based setting for emotional disorders beyond anxiety disorders. The focus of the present study was to establish the intervention’s efficacy and investigate its effects on symptomatology as well as satisfaction with life, positive and negative affect, negative effects, and treatment satisfaction. Since the UP also has clear postulations about underlying change processes, a previously published study focused on whether the intervention effects were mediated by transdiagnostic processes [28].

In the present study, we were interested in both overall and differential efficacy of the UP in the context of a guided self-help intervention for emotional disorders, namely anxiety, depressive, and somatic symptom disorders. We hypothesized that participants who received the Internet-based adaption of the UP would exhibit more improvement in symptom distress than participants in the waitlist group and that these effects would be maintained at 1- and 6-month follow-up. We further hypothesized that participants in the treatment group would shower greater changes over time in regard to the secondary outcomes, i.e. negative and positive affect, satisfaction with life and symptoms of anxiety and depression, than participants in the waitlist group. Finally, we hypothesized that these effects would also present in subgroups of participants with a primary anxiety, depressive, or somatic symptom disorder.

## Method

### Design

We compared the Internet-based intervention based on the UP to waitlist control in an RCT. The trial was registered under DRKS00014820 at DRKS. Participation was free of charge and not reimbursed.

### Ethics

The trial was approved by the ethics committee of the Department of Education and Psychology at Freie Universitaet Berlin, Germany (186/2018). There was a deviation from the study protocol in regard to sample size: As outlined in the trial study protocol, we had originally planned to conduct three separate studies on the Internet-based UP for anxiety, depressive, and somatic symptom disorders. Instead, we decided to investigate the differential efficacy for all three diagnostic groups within the current trial. Thus, sample size differed and block randomization was used to ensure balanced distribution of participants with an anxiety, depressive, or somatic symptom disorder across treatment and waitlist.

### Participants

We included participants if they (a) were over 18 years, (b) had a sufficient knowledge of German, (c) had Internet access, (d) had a stable dose of medication over the preceding three months, and (e) had one of the following primary diagnoses: panic disorder, agoraphobia, social anxiety disorder, generalized anxiety disorder, persistent depressive disorder, major depressive disorder, illness anxiety disorder, and somatic symptom disorder. We excluded participants from the study if they (a) currently experienced symptoms of a psychotic, bipolar, or substance use disorder, (b) were suicidal, or (c) were currently in psychotherapy.

We recruited participants in mental health online forums as well as via online advertising. Participants were directed to a study website with detailed information as well as a registration and consent form. After obtaining written informed consent, we activated an online screening with demographic and self-report measures. If participants’ ratings reached one or more pre-defined cut-offs (see below), we invited them to a structured clinical interview via telephone to determine inclusion and exclusion diagnoses [29].

Sample size was calculated using g*power [30]. We hypothesized a large effect (*Cohen’s d* = 0.8) in favor of the intervention [18, 24]. To detect this effect (one-sided t-test for independent samples, α = .05) with a power of 80%, a sample size of *n* = 42 participants is required. With an assumed attrition rate of 15%, we aimed at recruiting a sample size of *n* = 60 per diagnostic group (anxiety, depressive, and somatic symptom disorder).

### Intervention

The 10-week guided transdiagnostic intervention is an adaptation of the UP for Internet-based use. The guided self-help format requires Internet-based treatment to be comparatively shorter and more concise than face-to-face treatments. Other transdiagnostic Internet-based interventions range between five and ten modules [10]. We condensed and simplified the protocol to include a maximum of 10 modules, while retaining the core concepts of the UP (see Table 1). The sequence and contents of the modules were fixed for all participants. We recommended participants to complete one module per week, but participants were free to work at their own pace. Weekly asynchronous guidance was provided by the first and last author as well as by 16 graduate students of clinical psychology. The master students were trained in a 1-day workshop and biweekly supervision meetings were held throughout the trial to ensure adherence. On average, online therapists spent 24.44 (*SD* = 7.92) minutes per week on every participant.

**Table 1:** Overview of guided intervention.

Week	Name	Description
1	Motivation and goal setting	Participants learn about motivation and goal setting. They fill in a decisional balance sheet and set 1–3 goals for treatment.
2	Understanding Emotions	Participants learn about emotions and the difference between cognition, emotion, and behavior. They record their emotional experiences and reflect the short- and long-term consequences of their behavior.
3	Mindfulness	Participants learn about primary and secondary emotions and the benefits of mindful emotion awareness. They practice mindfulness with a selection of mindfulness exercises.
4	Cognitive Flexibility I	Participants learn about the relationship of thoughts and emotions, automatic thoughts and thinking traps. They challenge their thoughts by finding alternative thoughts.
5	Cognitive Flexibility II	Participants learn about thoughts about emotions and core beliefs. They continue with challenging their thoughts.
6	Countering Avoidance	Participants learn about avoidance and countering avoidance. They reflect on their avoidance tendencies and record their experience with countering avoidance.
7	Interoceptive Exposure	Participants learn about the effect of physical sensations. They induce physical sensations with video-guided interoceptive exposures and own exercises.
8	Emotion Exposure	Participants set a hierarchy of “difficult situations” and conduct emotion exposures in vivo and sensu.
9	Emotion Exposure II	Participants continue to expose themselves to emotion-inducing situations and images.
10	Relapse Prevention	Participants reflect on achievements and compile a training schedule for when treatment is over.

### Outcome measures

We interviewed participants with a structured clinical interview via telephone prior to randomization. After 10 weeks, participants of the treatment group were interviewed again to determine diagnostic status at post-treatment. All other outcomes were self-reported on the online platform. We assessed the primary outcome measure (BSI-18) and transdiagnostic secondary outcome measures at baseline, mid- (5 weeks) and post-treatment (10 weeks) as well at 1-month and 6-month follow-up after treatment completion. We assessed all disorder-specific measures at baseline and post-treatment (10 weeks). Negative effects and treatment satisfaction were collected at post-treatment (10 weeks). We chose commonly used and accepted self-report symptom measures whose psychometric qualities were evaluated for English and German versions and showed satisfactory psychometric properties in clinical and non-clinical groups. To relieve measurement burden on participants, we selected brief measures.

#### Structured clinical interview

We interviewed participants with the Diagnostic Interview for Mental Disorders (DIPS) [29, 31], a structured clinical interview for DSM-5, via telephone to determine diagnostic status of participants. The DIPS has good psychometric properties [29]. Interviewers were the first author as well as six master students of clinical psychology who were trained and supervised. Ten interviews were audiotaped and rated by an independent second rater. Interrater reliability ranged between 95.12% (anxiety disorders) and 100% accordance (all other diagnostic groups).

#### Primary outcome

Primary outcome was symptom distress at post-treatment, as measured by the Brief Symptom Inventory 18 (BSI-18). The BSI-18 [32, 33] is an 18-item short-version of the Symptom-Checklist-90-R and includes three subscales with 6 items each (anxiety, depression, and somatization). All items are rated on a 5-point Likert scale (0 = not at all to 4 = very strong). The total sum score, ranging from 0 to 72, serves as a global severity index. The BSI-18 shows high internal reliability (α = .91-.93) and overall satisfactory psychometric properties across clinical and non-clinical groups [33–36].

#### Transdiagnostic secondary outcome measures

We assessed positive and negative affect with the Positive and Negative Affect Schedule (PANAS). The PANAS [37, 38] measures positive and negative affect with two 10-item scales. Items are rated from 1 (very slightly or not at all) to 5 (extremely), with a maximum score of 50 for each subscale. Both scales show high internal consistency (α = .84-.90), satisfactory convergent and discriminant validity, and are quasi-independent. Satisfaction with life was assessed with the Satisfaction with Life Scale (SWLS). The SWLS [39, 40] is widely used and economic 5-item measure of life satisfaction, rated on a 7-point Likert scale (1 = strongly disagree to 7 = strongly agree). Internal consistency (α = 0.89–0.92) and convergent and divergent validity are satisfactory [41].

#### Disorder-specific secondary outcome measures

We assessed depressive symptoms with the Patient Health Questionnaire (PHQ-9). The PHQ-9 [42, 43] is an internally reliable (α = 0.88) 9-item screening instrument for depression. Nine symptoms of depression (e.g., “feeling tired or having little energy”) are rated on a 4-point Likert scale (1 = not at all present to 4 = present nearly every day). Symptoms of generalized anxiety were assessed with the 7-item Generalized Anxiety Disorder Screener (GAD-7) [44, 45]. The seven items (e.g., “not being able to stop or control worrying”) are rated on a 4-point Likert scale (0 = not at all present to 3 = present nearly every day). The GAD-7 is unidimensional and internally reliable (α = 0.92). The Liebowitz Social Anxiety Schedule (LSAS) was used to assess social anxiety. The LSAS [46, 47] is 24-item measure of social anxiety assessing fear/anxiety and avoidance of social situations. Internal consistency of the two subscales as well as the total scale is high (α = 0.82–0.95) and the scale exhibits good convergent and discriminant validity [48]. We assessed panic and agoraphobia with the 13-item Panic and Agoraphobia Scale (PAS) [49, 50]. The PAS assesses panic attacks and agoraphobic avoidance as well as related concerns on a 5-point Likert-scale (0 to 4). Internal reliability (α = 0.88) as well as psychometric properties are good. Health anxiety was assessed with the 18-item Short Health Anxiety Inventory (SHAI) [51, 52]. The SHAI has a two-factor structure and assesses health anxiety with 14 items and perceived negative consequences of being ill with four items. For each item, participants select one of four statements which are scored from 0 to 3. The SHAI scales show high internal reliability (α = .83-.97) and have good psychometric properties. We used the 15-item Patient Health Questionnaire (PHQ-15) to assess somatic symptom burden [53, 54]. The scale comprises 15 of the most common somatic symptoms, each scored from 0 (= not bothered at all) to 2 (= bothered a lot). Internal consistency (α = 0.80) and other psychometric properties are good. Participants were invited to the structured clinical interview if their baseline score exceeded one of the following disorder-specific outcome measures: PHQ-9 > 10 [55], LSAS > 30 [56], GAD-7 >10 [44], PAS > 9 [50], or SHAI > 18 [51].

#### Negative effects and satisfaction with treatment

We assessed negative effects of treatment with the Negative Effects Questionnaire (NEQ) [57], a 20-item measure of the occurrence and characteristics of negative effects following treatment. Each item is rated in three steps: if participants agree that the negative effect occurred, then they are asked to rate the negative impact of the negative effect and attribute it to treatment or other circumstances. We used the Client Satisfaction Questionnaire-8 (CSQ-8) [58, 59] to measure client satisfaction with treatment with 8 questions. The CSQ-8 is unidimensional and internally reliable [60].

#### Treatment adherence, usage & attrition

We based our definition of completers on the number of modules completed. We defined completer status as having completed six or more sessions within ten weeks, which is equal to being exposed to all core elements of therapy (mindfulness, cognitive flexibility, and emotion avoidance). Non-completers completed five or less sessions within ten weeks and/or communicated treatment termination. The following usage metrics were routinely collected by the online program: number of logins, time spent on the platform, number of completed exercises and written messages. Time spent on the platform does not reflect actual usage time, as the website did not automatically log participants out. We defined attrition as the percentage of participants who failed to complete post-treatment outcome measures.

### Randomization procedure

We randomized participants to waitlist or treatment in a 1:1 ratio. We used stratified block randomization to ensure a balanced distribution of primary diagnosis groups (anxiety, depressive, and somatic symptom disorder as stratum) across treatment and waitlist. The allocation sequence was generated by an online random number generator and carried out by the online platform that also hosted the intervention.

### Statistical analysis

We ran all analyses in RStudio [61]. All analyses followed the intent-to-treat framework, i.e. all randomized participants were included in the analyses [62]. The intention-to-treat analyses for the efficacy and follow-up analyses were conducted with linear mixed effects models with maximum likelihood estimation to handle missing data. We fitted linear mixed effects models with the lmer-function from the lme4 package [63], using the lmerTest package to obtain p-values [64]. For the efficacy analysis, we included treatment, time, and the interaction of treatment and time as fixed and participants as random effects in the model. We also conducted the same analysis in a subgroup of primarily anxious and primarily depressed participants. To analyze whether treatments gains were maintained in the treatment group at 1- and 6-month follow up, we included time as fixed and participants as random effects in the model. Statistical significance was set at the 5% level, and confidence intervals were calculated at the 95% confidence level. We calculated effect sizes (*Hedges’ g*) based on the estimated means and the pooled standard deviations of the observed means. Response was determined based on the reliable change index (*RCI* = 1.96) [65]. RCIs were calculated using the BSI-18’s internal reliability (*α* = .91) [33, 66]. Since norm data from a clinical and general population was available [33, 36], we also calculated the *Cutoff C* to determine recovery rates [65]. Individuals are recovered if they fall below the cutoff and show reliable improvement according to the RCI [65].

## Results

### Participants

**Fig 1:**
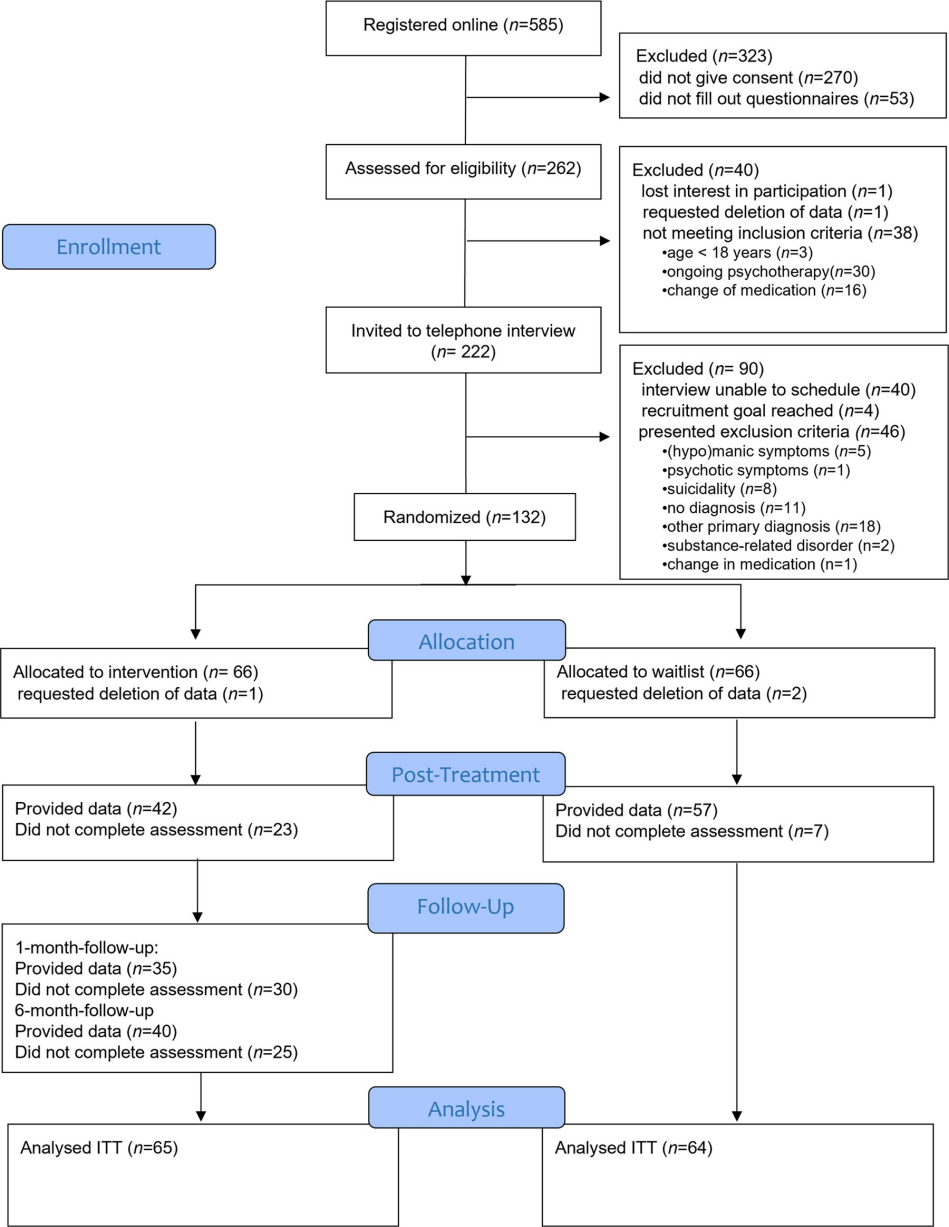
Consort flow chart. *Note*. Since this present study, which focuses on establishing efficacy, and a previously published study, which focuses on underlying mechanisms of change [28], are based on the same RCT, the participant flow charts of both studies are identical.

A detailed description of the recruitment procedure is shown in the flow diagram in Fig 1. Recruitment commenced in December 2018 and ended in February 2020. A total of *n* = 132 participants were randomized: *n* = 60 with a primary anxiety, *n* = 60 with a primary depressive disorder, and *n* = 12 with a primary somatic symptom disorder. Despite increased efforts, we failed to meet our original recruitment goal regarding somatic symptom disorder within the study’s timeframe. Demographic characteristics are displayed in Table 2. Groups did not differ in demographic variables.

**Table 2:** Demographic characteristics of participants at baseline.

				Statistical test of group difference		
Demographic Variable	Treatment (*n* = 65)	Waitlist (*n* = 64)	Total	df	*χ2 / t*	*p*
**Gender**				1	.48	.49
** Male**	23 (35.4%)	18 (28.1%)	41 (31.8%)			
** Female**	42 (64.6%)	46 (71.9%)	88 (68.2%)			
**Age**				127	.18	.86
** Mean (SD)**	37.51 (11.99)	37.11 (13.04)	37.31 (12.47)			
** Range**	18–67	18–66	18–67			
**Relationship**				1	1.99	.16
** in a relationship**	40 (61.5%)	30 (46.9%)	70 (54.3%)			
** single**	25 (38.5%)	34 (53.1%)	59 (45.7%)			
**Highest Education**				4	4.7	.32
** up to 9 years of school education**	4 (6.2%)	5 (7.8%)	9 (7%)			
** Secondary school**	10 (15.4%)	11 (17.2%)	21 (16.3%)			
** College entrance qualification**	16 (24.6%)	25 (39.1%)	41 (31.8%)			
** College / university degree**	34 (52.3%)	22 (34.4%)	56 (43.3%)			
** other**	1 (1.5%)	1 (1.6%)	2 (1.6%)			
**Employment status**				5	2.99	.7
** employed**	33 (50.8%)	29 (45.3%)	62 (48.1%)			
** self-employed**	4 (6.2%)	5 (7.8%)	9 (7%)			
** in education**	12 (18.5%)	17 (26.6%)	29 (22.5%)			
** pensioned**	3 (4.6%)	5 (7.8%)	8 (6.2%)			
** unemployed**	9 (13.8%)	6 (9.4%)	15 (11.6%)			
** other**	4 (6.2%)	2 (3.1%)	6 (4.7%)			
**Currently on medication**				1	0	.99
** yes**	16 (24.6%)	16 (25%)	32 (24.8%)			
** no**	49 (75.4%)	48 (75%)	97 (75.2%)			
**Prior Psychotherapy**				1	1.95	.16
** yes**	44 (67.7%)	51 (79.7%)	95 (73.6%)			
** no**	21 (32.3%)	13 (20.3%)	34 (26.4%)			
**Primary Diagnosis**				9	5.66	.77
** Agoraphobia**	3 (4.6%)	4 (6.3%)	7 (5.4%)			
** Generalized Anxiety Disorder**	9 (13.8%)	5 (7.8%)	14 (10.8%)			
** Panic Disorder**	5 (7.7%)	5 (7.8%)	10 (7.8%)			
** Social Anxiety Disorder**	13 (20%)	16 (25%)	29 (22.5%)			
** Major Depressive Disorder**	14 (21.5%)	17 (26.6%)	31 (24%)			
** Persistent Depressive Disorder**	15 (23.1%)	12 (18.8%)	27 (20.9%)			
** Somatic Symptom Disorder**	2 (3.1%)	3 (6.3%)	5 (3.9%)			
** Illness Anxiety Disorder**	4 (6.2%)	2 (3.1%)	6 (4.7%)			
**Comorbidity**						
** 1 comorbid diagnosis**	51 (78.5%)	48 (75%)	99 (76.7%)			
** 2 comorbid diagnoses**	27 (41.5%)	28 (43.7%)	55 (42.6%)			
** 3 comorbid diagnoses**	18 (27.7%)	18 (28.1%)	36 (27.9%)			
** 4 comorbid diagnoses**	12 (18.5%)	10 (15.6%)	22 (17.1%)			
** 5 comorbid diagnoses**	5 (7.7%)	4 (6.2%)	9 (6.9%)			

*Note*. Since this present study, which focuses on establishing efficacy, and a previously published study, which focuses on underlying mechanisms of change [28], are based on the same sample, the descriptive characteristics of both studies are identical.

### Treatment adherence, usage & attrition

Following our definitions of treatment completers (>6 modules), *n* = 47 (73.4%) participants were completers and *n* = 17 (26.6%) were non-completers. However, only *n* = 27 (42.2%) completed all 10 modules. Participants on average logged in 85.3 (*SD* = 39.2) times and spent 57.4 (*SD* = 34.9) hours on the platform. However, this does not reflect actual usage time, as the website did not automatically log participants out. Participants completed on average 7.22 (*SD* = 3.15) modules, 36.17 (*SD* = 29.04) exercises, 0.41 (*SD* = 0.3) exercises per login and wrote 8.05 (*SD* = 7.97) messages. In the treatment group, *n* = 23 (35.38%) failed to complete post-treatment questionnaires, regardless of how they adhered to treatment. In the waitlist group, *n* = 7 (10.94%) failed to complete assessments after 10 weeks of waiting. For the follow-up assessments, *n* = 30 participants of the treatment group provided data at 1-month follow-up and *n* = 40 participants at 6-month follow-up.

### Efficacy and maintenance of treatment effects

Participants in the treatment group showed greater changes in primary and secondary outcomes over time than participants in the waitlist (see Table 3). Predicted means, standard deviations, and effect sizes (*Hedges’ g*) are displayed in Table 4. Between-group effect sizes ranged between *g* = 1.12 (symptom distress) and *g* = 0.20 (health anxiety) and within group effect sizes ranged between *g* = 1.38 (symptom distress) and *g* = 0.32 (social anxiety). Treatment gains for the transdiagnostic outcome measures—symptom distress (BSI-18), positive and negative affect (PANAS), as well as satisfaction with life (SWLS)—were maintained at 1- and 6-month-follow-up (*B* = -1.5–0.44, *t* = -1.34–0.67, *df* = 68.12–73.0, *p* > .05).

**Table 3:** Results from the linear mixed models for the interaction effect (time x group) for primary and secondary outcome measures (pre-post).

	*B*	*SE*	*df*	*t*	*p*
BSI-18	9.16	1.6	198.48	5.70	<0.01
PANAS NA	4.48	1.29	200.46	3.46	<0.01
PANAS PA	-3.90	1.23	206.59	-3.17	<0.01
SWLS	-2.52	0.75	191.15	-3.36	<0.01
PHQ-9	3.89	0.96	107.36	4.05	<0.01
GAD-7	3.13	0.84	104.66	3.75	<0.01
LSAS	6.63	3.08	100.08	2.15	0.03
PAS	5.43	1.91	102.85	2.85	<0.01
SHAI	4.14	1.14	96.08	3.62	<0.01
PHQ-15	2.07	0.86	101.88	2.41	0.02

*Note*. BSI-18: Brief Symptom Inventory 18. PANAS NA: Negative Affect Subscale of the Positive and Negative Affect Schedule. PANAS PA: Positive Affect Subscale of the Positive and Negative Affect Schedule. SWLS: Satisfaction with Life Scale. PHQ-9: Patient Health Questionnaire 9. GAD-7: Generalized Anxiety Screener 7. LSAS: Liebowitz Social Anxiety Scale. PAS: Panic and Agoraphobia Scale. SHAI: Short Health Anxiety Inventory. PHQ-15: Patient Health Questionnaire

**Table 4:** Predicted means and standard errors for primary and secondary outcome measures and within- and between-group effect sizes.

Measure		Treatment		Waitlist		Pre-Post Within ES *Hedges’ g* 95% CI				Between group ES *Hedges’ g* 95% CI	
		*M*	*SE*	*M*	*SE*	Treatment	Waitlist				
BSI-18	Baseline	25.8	1.29	26.62	1.15	-1.38		-0.36		-1.11	
	10 weeks	13.11	1.37	23.09	1.25	[-1.77, -1]		[-0.71, -0.01]		[-1.48,-0.74]	
	1-month FU	14.20	1.37								
	6-month FU	12.58	1.31								
PANAS NA	Baseline	27.38	0.99	28.2	0.82	-0.94		-0.34		-0.76	
	10 weeks	20.54	1.04	25.81	0.95	[-1.3, -0.58]		[-0.69, 0]		[-1.11, -0.4]	
	1-month FU	20.62	1.06								
	6-month FU	20.20	1.02								
PANAS PA	Baseline	20.11	0.64	19.03	0.62	0.79		0.3		0.70	
	10 weeks	25.63	0.91	20.61	0.81	[0.43, 1.14]		[-0.05, 0.65]		[0.35, 1.06]	
	1-month FU	25.35	1.24								
	6-month FU	24.86	1.19								
SWLS	Baseline	15.64	0.74	15.45	0.77	0.46		0.07		0.39	
	10 weeks	18.57	0.85	15.9	0.81	[0.11, 0.81]		[-0.28, 0.42]		[0.05, 0.74]	
	1-month FU	19.22	1.01								
	6-month FU	19.27	1.0								
PHQ-9	Baseline	13.86	0.6	14.67	0.62	-1.15		-0.34		-0.91	
	10 weeks	8.2	0.74	12.9	0.66	[-1.52, -0.78]		[-0.69, 0.01]		[-1.27, -0.55]	
GAD-7	Baseline	11.77	0.61	12.42	0.52	-1.01		-0.32		-0.85	
	10 weeks	7.18	0.67	10.97	0.59	[-1.37, -0.64]		[-0.67, 0.0]		[-1.21, -0.49]	
LSAS	Baseline	58.52	3.32	69.23	3.58	-0.32		-0.06		-0.69	
	10 weeks	50.33	3.59	67.67	3.4	[-0.67, 0.03]		[-0.4, 0.29]		[-1.04, -0.33]	
PAS	Baseline	18.43	1.59	18.19	1.41	-0.64		-0.21		-0.49	
	10 weeks	10.72	1.64	15.9	1.49	[-0.99, -0.29]		[-0.56, 0.13]		[-0.84, -0.14]	
SHAI	Baseline	41.82	1.25	39.72	1.26	-0.57		-0.12		-0.20	
	10 weeks	36.41	1.36	38.45	1.3	[-0.92, -0.22]		[-0.47, 0.23]		[-0.55, 0.14]	
PHQ-15	Baseline	12.06	0.65	13.28	0.67	-0.65		-0.25		-0.61	
	10 weeks	8.62	0.76	11.91	0.69	[-1.0, -0.3]		[-0.6, 0.1]		[-0.97, -0.26]	

*Note*. FU: follow-up. ES: Effect Size. BSI-18: Brief Symptom Inventory 18. PANAS NA: Negative Affect Subscale of the Positive and Negative Affect Schedule. PANAS PA: Positive Affect Subscale of the Positive and Negative Affect Schedule. SWLS: Satisfaction with Life Scale. PHQ-9: Patient Health Questionnaire 9. GAD-7: Generalized Anxiety Screener 7. LSAS: Liebowitz Social Anxiety Scale. PAS: Panic and Agoraphobia Scale. SHAI: Short Health Anxiety Inventory. PHQ-15: Patient Health Questionnaire

### Subgroup analyses

Means, standard deviations and effect sizes (*Hedges’ g*) for primary and secondary outcome measures in the anxiety, depression, and somatic symptom disorder subsample are displayed in Table 5. For the anxiety subsample, the interaction of time x group was significant for symptom distress, positive affect, satisfaction with life, panic/agoraphobia, and depression (*B* = -4.75–8.71, *SE* = 1.24–2.86, *t* = -2.77–3.44, *df* = 42.70–87.42, *p* < 0.05), but not for negative affect, generalized anxiety, social anxiety, health anxiety, and somatization (*B* = 1.95–3.03, *SE* = 1.41–4.76, *t* = 0.54–1.92, *df* = 40.6–85.83, p >0.05). For the depressive subsample, the interaction of time x group was significant for symptom distress, negative affect, depression, generalized anxiety, social anxiety, and health anxiety (*B* = 3.15–11.01, *SE* = 1.13–4.61, *t* = 2.39–4.00, *df* = 43.95–88.49, *p* < 0.05), but not for positive affect, satisfaction with life, panic/agoraphobia and somatization (*B* = -3.27–2.24, *SE* = 0.94–2.58, *t* = -1.83–1.38, *df* = 45.44–88.29, *p* > 0.05). Due to small sample size, the somatic symptom disorder sample was not analyzed separately.

**Table 5:** Means in primary anxiety, depressive and somatic symptom disorders subsamples and effect sizes in anxious and depressive subsample.

		Anxiety Disorders						Depressive Disorders						Somatic Symtom Disorders			
		Treatment		Waitlist		Between group ES	Within group ES	Treatment		Waitlist		Between group ES	Within group ES	Treatment		Waitlist	
		*M*	*SE*	*M*	*SE*	*Hedges‘ g* 95% CI	*Hedges‘ g* 95% CI	*M*	*SE*	*M*	*SE*	*Hedges‘ g* 95% CI	*Hedges‘ g* 95% CI	*M*	*SD*	*M*	*SD*
BSI-18	Baseline	25.63	1.97	28.48	1.85	-1.09 [-1.46, -0.72]	-1.27 [-1.65, -0.9]	25.31	1.91	25.27	1.39	-1.08 [-1.45, -0.71]	-1.28 [-1.65, -0.9]	29.0	9.57	24.0	13.13
	10 weeks	13.14	2.34	24.71	2.09			13.69	1.8	22.4	1.65			10.0	4.90	18.2	5.22
PANAS NA	Baseline	28.21	1.48	30.72	1.31	-0.65 [-1.01, -0.3]	-0.86 [-1.22, -0.5]	26	1.45	26.5	.92	-0.85 [-1.21, -0.49]	-0.89 [-1.25, -0.53]	30.6	7.64	23.8	6.61
	10 weeks	21.76	1.62	26.54	1.43			19.46	1.47	25.42	1.35			19.8	5.26	23.6	5.9
PANAS PA	Baseline	21.4	1.02	21.14	0.98	0.69 [0.33, 1.04]	0.78 [0.42, 1.13]	18.1	0.76	17.1	0.76	0.58 [0.23, 0.94]	0.8 [0.44, 1.15]	24.0	4	18.4	3.65
	10 weeks	26.79	1.44	21.78	1.25			23.76	1.23	19.49	1.11			29.6	3.51	20.6	4.83
SWLS	Baseline	14.79	1.16	15.83	1.21	0.28 [-0.07, 0.63]	0.58 [0.23, 0.93]	15.62	1.02	14.1	0.79	0.52 [0.17, 0.87]	0.3 [-0.04, 0.65]	19.83	5.34	21.4	10.33
	10 weeks	18.22	1.32	16.47	1.22			17.65	1.12	14.18	1.04			25.2	3.42	22.8	9.04
PHQ-9	Baseline	12.83	0.9	13.59	1.11	-0.92 [-1.29, -0.56]	-1.17 [-1.54, -0.8]	14.97	0.8	16.37	0.56	-0.99 [-1.35, -0.62]	-1.16 [-1.53, -0.79]	13.67	6.5	10.8	3.83
	10 weeks	7.4	1.25	12.2	1.07			9.44	0.92	14.27	0.83			5.0	5.1	9	4.18
GAD-7	Baseline	11.77	0.95	13.97	0.71	-1.11 [-1.48, -0.74]	-1.01 [-1.38, -0.65]	11.07	0.92	10.97	0.69	-0.7 [-1.06, -0.35]	-0.93 [-1.29, 0.56]	15.17	2.32	12.2	6.14
	10 weeks	7.07	1.07	11.97	0.89			6.94	0.93	9.99	0.84			8.4	5.5	11.2	4.82
LSAS	Baseline	66.7	4.7	75.41	6.04	-0.45 [-0.8, -0.1]	-0.32 [-0.66, 0.03]	57	4.32	67.3	4.35	-0.93 [-1.3, -0.57]	-0.47 [-0.82, -0.12]	25.0	23.63	45	19.29
	10 weeks	59.14	5.7	70.41	5.34			46.72	4.66	68.02	4.3			26.8	33.01	49	13.17
PAS	Baseline	22.9	2.31	24	1.99	-0.6 [-0.95, -0.25]	-0.8 [-1.15, -0.44]	12.59	2.06	14.07	1.67	-0.4 [-0.74, -0.05]	-0.37 [-0.71, -0.02]	24.33	11.11	9.2	10.64
	10 weeks	12.65	2.55	19.66	2.26			8.78	2.06	12.5	1.9			11.75	6.8	14	8.72
SHAI	Baseline	42.13	1.62	41.62	1.99	-0.24 [-0.58, 0.11]	-0.54 [-0.89, -0.19]	38.31	1.61	36.2	1.55	-0.25 [-0.6, 0.1]	-0.64 [-1, -0.29]	57.17	8.38	49.8	6.38
	10 weeks	36.99	2.09	39.51	1.92			33.52	1.68	35.55	1.6			45.75	7.89	49	12.31
PHQ-15	Baseline	12.5	0.98	14.21	0.97	-0.69 [-1.05, -0.34]	-0.82 [-1.17, -0.46]	11.21	0.93	12.83	1.05	-0.51 [-0.86, -0.16]	-0.41 [-0.75, -0.06]	14.0	5.97	10.6	3.29
	10 weeks	8.34	1.22	11.99	1.05			8.96	1.02	11.87	1.08			8.75	4.99	11	4.95

*Note*. ES: Effect Size. BSI-18: Brief Symptom Inventory 18. PANAS NA: Negative Affect Subscale of the Positive and Negative Affect Schedule. PANAS PA: Positive Affect Subscale of the Positive and Negative Affect Schedule. SWLS: Satisfaction with Life Scale. PHQ-9: Patient Health Questionnaire 9. GAD-7: Generalized Anxiety Screener 7. SAS: Liebowitz Social Anxiety Scale. PAS: Panic and Agoraphobia Scale. SHAI: Short Health Anxiety Inventory. PHQ-15: Patient Health Questionnaire 15. For the somatic symptom disorder subsample only observed means and standard deviations are reported because of small sample size.

### Response and recovery rates

The majority of participants *(n* = 29; 69%) in the treatment group reliably improved, while *n* = 13 (31%) showed no reliable change. Nobody deteriorated. If participants who did not complete post-treatment assessments were classified as non-responders, the ratio of responders and non-responders would shift to 55% non-responders and 45% responders. Approximately half of the participants (*n* = 20; 48%) reliably recovered (falling below the *Cutoff C* and depicting reliable change).

### Satisfaction with treatment and negative effects

Overall, 84.62% of the participants in the intervention group were “mostly” or “very” satisfied with treatment. Mean satisfaction was 3.27 out of 4 (*SD* = 0.62).

79.49% of participants in the treatment group reported at least one negative treatment effect. Participants reported on average 2.54 negative treatment effects (*SD* = 2.78). “Unpleasant memories resurfaced” (38.46%) and “I experienced more unpleasant feelings” (38.46%) showed the highest frequency. Items associated with elevated symptoms (“symptom factor”) showed the highest mean (*M* = 2.31, *SD* = 3.18). Participants felt that the negative effects affected their well-being slightly to moderately (*M* = 1.39, *SD* = 0.58).

### Diagnostic status

We reached *n* = 37 (57.81%) of the treatment group for the post-treatment structured clinical interview. The mean number of total diagnoses dropped to 0.81 (*SD* = 1.08). The majority of participants did not fulfill criteria for their primary diagnosis (*n* = 26; 70.27%). In regard to comorbidity, *n* = 24 (64.86%) did not fulfill criteria for comorbid diagnoses. The number of comorbid diagnoses at post-intervention ranged from 0 to 3. If participants who we did not reach for the post-treatment interview experienced no effect on their primary diagnosis, 59.3% of participants would be unchanged, while 40.6% of participants did not fulfill criteria of their primary diagnosis.

## Discussion

This study investigated the efficacy of a guided Internet-based transdiagnostic intervention based on the UP. Overall, participants who received the intervention showed greater changes in symptom distress, positive and negative affect, life satisfaction, as well as symptoms of anxiety and depression over time than participants in the waitlist group. The medium to large effects between treatment and waitlist group suggest that the intervention is effective across a range of symptoms in the internalizing spectrum and targets positive and negative affect. These results are comparable both to research on the UP’s efficacy as a face-to-face and Internet-based intervention, as well as other (transdiagnostic) Internet-based treatments [10, 18–20, 67]. The transdiagnostic intervention’s impact on comorbidity is of special interest. The mean number of diagnoses in the fairly comorbid sample (78% comorbidity) dropped from almost 3 (range 1–8) to under 1 (range 0–3) which is very comparable to other face-to-face trials on the UP [18, 68] and suggests that comorbidity can be effectively addressed in an Internet-based setting.

Our findings substantiate previous findings on the efficacy of applying the UP principles online. Besides Tulbure et al. (2018)’s investigation, two other studies investigated a transdiagnostic Internet-based treatment combining treatment principles from the UP and other emotion-regulation treatments, and found that this transdiagnostic Internet-based intervention reduces symptoms of depression, anxiety, and negative affect and enhances positive affect [20, 21].

In contrast, Tulbure et al. (2018)’s and our intervention were modeled more closely after the original UP. Although developed independently, both interventions were highly similar in length and content and produced comparable results. The degree of comorbidity, prior psychotherapy, and current medication was higher in our sample compared to the other Internet-based UP and we have not applied an upper limit for symptom severity [20]. Our results suggest that Internet-based interventions based on the UP can be expanded to comorbid and clinically more severe patient populations. Subgroup analyses also revealed that the intervention in our study produced similar effects in participants with primary anxiety or depression. While preliminary, these results are encouraging of the UP’s potential to treat depression. However, since the UP was condensed and simplified to fit the Internet-based treatment modality, differences in efficacy between Internet-based and face-to-face-applications of the treatment may be present. A future comparison of an Internet-based and a face-to-face version of the UP may shed light on differential effects in anxiety, depression, and other diagnostic groups.

Beyond symptom improvements, other indicators are important determining factors for the uptake of interventions. About 75% of participants completed the core modules of treatment. While this adherence rate is not unusual in Internet-based settings [69], higher adherence may have benefitted outcomes, especially considering that less than half of participants completed all modules [10]. Usage data suggested that participants who chose to work with the program engaged with it actively. The majority of participants was mostly satisfied to very satisfied with the program but also reported experiencing negative effects. That approximately 80% of participants report negative effects is higher than reports from other trials (56–65%) [57]. Negative effects are not synonymous to unwanted effects necessarily–that participants experience more negative emotions short-term can even be an intended effect in a program focused on reducing avoidance and experiencing emotions.

Our results need to be interpreted in the light of several limitations: The trial suffered from an attrition rate of 35% of participants who did not provide post-treatment data. High attrition rates like this are not uncommon in Internet-based treatments [70] but they can limit the validity and reliability of findings and could be indicative of limited acceptability of the intervention. Subgroup analyses of the anxiety and depression subsamples revealed that not all symptom measures showed significant effects, likely due to small sample sizes and limited statistical power related to dropout. We failed to recruit a sufficient sample of participants with somatic symptom disorders within the study’s timeframe. Recruitment via primary health care providers might be a more fruitful recruitment strategy for this population [e.g., 71]. The self-selection of participants and the predominantly female, highly educated, and psychotherapy-experienced sample limit the generalization of results.

Overall, these results strengthen the evidence base of the UP principles as a viable treatment option beyond anxiety disorders and demonstrate that the UP can successfully be delivered over the Internet over the course of ten weeks. Applying a transdiagnostic intervention online can help to overcome barriers to treatments and accelerate the dissemination of evidence-based treatments for a broad variety of the most common disorders. However, dropout and attrition rates suggest that several modifications should be made to the intervention before employing it, e.g. by further simplifying it, delivering it modularly [72, 73], or implementing monitoring to detect participants at risk of treatment failure [74]. While a waitlist control group is a necessary first step to establish efficacy, the intervention should next be measured against an active control condition, e.g. against disorders-specific treatments.

## Supporting information

S1 Checklist (DOC)

S1 File (DOCX)
