# Supplementary material for: The unified protocol as an internet-based intervention for emotional disorders: Randomized controlled trial
Source: PLoS One. 2022 Jul 11;17(7):e0270178. doi: 10.1371/journal.pone.0270178 (PMC9273095; doi:10.1371/journal.pone.0270178)
Supplement: S1 File — (DOCX) [file pone.0270178.s002.docx]

**Trial Protocol**

**… in German, as submitted to the Ethics Board of the Department of Education and Psychology, Freie Universität Berlin: p.2**

**… translated to English: p.24**

**Antrag auf Beratung durch die Ethikkommission der Freien Universität Berlin zur Durchführung einer wissenschaftlichen Studie**

Titel: Machbarkeit, Akzeptanz und Wirksamkeit der Online-Intervention iUP

Namen und Anschriften der beteiligten Wissenschaftlerinnen

Dr. Johanna Böttcher, Freie Universität Berlin, Fachbereich Erziehungswissenschaft und Psychologie, Arbeitsbereich Klinische Psychologie und Psychotherapie,
Habelschwerdter Allee 45, 14195 Berlin, Tel.: 030-838-56569, E-Mail: [johanna.boettcher@fu-berlin.de](mailto:b.renneberg@fu-berlin.de)

Dipl.-Psych. Carmen Schäuffele, Freie Universität Berlin, Fachbereich Erziehungswissenschaft und Psychologie, Arbeitsbereich Klinisch-Psychologische Intervention,

Habelschwerdter Allee 45, 14195 Berlin, Tel.: 030-838-63696 E-Mail: carmen.schaeuffele@fu-berlin.de

## Zusammenfassung des Antragsgegenstandes

Ziel der geplanten Studien ist die Untersuchung der Machbarkeit, Akzeptanz und Wirksamkeit der onlinebasierten Intervention „iUP“. iUP ist ein 10-wöchiges onlinebasiertes Therapieprogramm für Personen mit Angststörungen, Depressionen und Somatischen Belastungsstörungen. In diesem Programm lernen die Teilnehmer*innen wöchentlich neue Inhalte kennen und arbeiten anhand von Übungen an ihrem Umgang mit Emotionen. Es wird für das iUP eine geleitete und ungeleitete Version geben. In der geleiteten Version werden Teilnehmende von einem persönlichen Berater wöchentlich personalisiertes Feedback zu ihrer Arbeit im Programm erhalten. In der ungeleiteten Version erhalten die Teilnehmer*innen automatisiertes, standardisiertes Feedback. Es sind drei Studien geplant: In einer ersten Studie („Pilotstudie“) soll die geleitete Version des iUP an einer Stichprobe von Personen mit Angststörungen (sozialer Angststörung, generalisierter Angststörung, Panikstörung, Agoraphobie), Depressionen und Somatischen Belastungsstörungen (Somatische Belastungsstörung mit vordergründiger Krankheitsangst und Krankheitsangststörung) evaluiert werden. In einer zweiten Studie wird die Wirksamkeit der geleiteten Version für Menschen mit primär depressiven Symptomen untersucht und mit einer anderen aktiven Behandlung verglichen. In der dritten Studie wird die Wirksamkeit des ungeleiteten iUPs für Menschen mit Krankheitsängsten näher untersucht und mit einem ungeleiteten Achtsamkeitsprogramm verglichen.

## Ziel der Studien

Die sogenannten emotionalen Störungen, zu denen die Angst-, depressiven und Somatischen Belastungsstörungen zählen, haben eine hohe Prävalenz in der Allgemeinbevölkerung und hohe Komorbiditätsraten (Jacobi et al., 2014). Die Komorbiditätsraten sind nicht nur für die Betroffenen belastend, sondern stellen auch für die Behandlung eine Herausforderung dar. Klassische verhaltenstherapeutische Manuale fokussieren nur auf ein Störungsbild und geben meist keinen Hinweis darauf, wie mit komorbiden Symptomen umgegangen werden soll. Um mehrere psychische Störungen zeitgleich in der Therapie zu berücksichtigen wurden sogenannte transdiagnostische Therapieansätze entwickelt (Harvey, 2004; Mansell, Harvey, Watkins, & Shafran, 2009). Das Unified Protocol ist ein transdiagnostisches Behandlungsprogramm, das alle emotionalen Störungen anspricht (Barlow et al., 2018). Das Unified Protocol wurde in den USA face-to-face in mehreren Studien evaluiert und es finden sich vor allem für die Angststörungen Hinweise auf seine Wirksamkeit (Barlow et al., 2017; Farchione et al., 2012). Die Wirksamkeit für depressive Störungen wurde bisher nur in Einzelfalluntersuchungen untersucht (Boswell, Anderson, & Barlow, 2014). Auch für andere emotionale Störungen wie die Somatische Belastungsstörung und die Krankheitsangststörung fehlen Befunde zur Wirksamkeit des Unified Protocols.

Die Adaptation des Unified Protocols in einem Onlinekontext würde es erlauben, komorbide emotionale Störungen gleichzeitig zu behandeln und dabei die Vorteile der Onlinetherapie zu nutzen. Zahlreiche Studien und Meta-Analysen belegen die Wirksamkeit von onlinebasierter Therapie im Bereich der Depressionen, Angststörungen und Krankheitsängste (Andersson & Cuijpers, 2009; Andrews, Cuijpers, Craske, McEvoy, & Titov, 2010; Barak, Hen, Boniel-Nissim, & Shapira, 2008; Cuijpers et al., 2009; Hedman et al., 2011; Hedman, Axelsson, Andersson, Lekander, & Ljótsson, 2016; Newby, Mahoney et al., 2016). Online-Interventionen bieten Betroffenen einen leichten, barrierefreien, flexiblen und relativ anonymen Zugang zu evidenz-basierter Therapie (Andersson & Titov, 2014).

In den geplanten Studien soll daher eine **I**nternet-basierte Version des **U**nified **P**rotocols (iUP) evaluiert werden. Um iUP im deutschen Sprachraum zugänglich zu machen, wird das englische Therapiemanual übersetzt und an Patient*innen mit einer oder mehreren emotionalen Störungen in drei Studien auf Machbarkeit, Akzeptanz und Effektivität erprobt.

**Studie 1:** Die Pilotstudie untersucht die Machbarkeit, Akzeptanz und Effektivität der geleiteten Version mit einem randomisiert-kontrollierten Design an einer Stichprobe von Patient*innen mit Angststörungen, depressiven und somatischen Belastungsstörungen. Teilnehmer*innen, die der Warteliste-Kontrollgruppe zugewiesen werden, erhalten nach zehn Wochen Zugang zur Intervention.

**Studie 2:** Wie erwähnt fehlen Studien zur Wirksamkeit des UPs bei Depressionen. Das Ziel der zweiten Studie ist es deshalb, das transdiagnostische iUP in der geleiteten Version an einer Stichprobe von depressiven Patienten zu untersuchen und mit einer depressionsspezifischen geleiteten Online-Intervention (TK-DepressionsCoach) zu vergleichen. Der TK-DepressionsCoach ist eine an der Freien Universität in Kooperation mit der Techniker Krankenkasse entwickelte geleitete sechswöchige depressionsspezifische Online-Intervention. Ähnlich wie in der geplanten geleiteten iUP-Intervention lernen Teilnehmer*innen psychoedukative Inhalte kennen und arbeiten eine Woche lang an einer Hausaufgabe bis sie personalisiertes Feedback erhalten. In einer ersten Studie zum TK-Depressionscoach mit N=1089 Teilnehmer*innen zeigten sich sehr große within-group Effekte für die depressive Symptomatik (d=1.20) (Zagorscak, Heinrich, Sommer, Wagner, & Knaevelsrud, 2018). Auch aktuell läuft eine Studie zum TK-DepressionsCoach mit bereits mehr als 1000 Teilnehmern. Wir möchten in Studie 2 die große Stichprobe des TK-DepressionsCoach nutzen um mithilfe von Matched Sampling eine Kontrollgruppe für das iUP zu konstruieren (z.B. Rosenbaum & Rubin, 1985). Das Matching der zwei Gruppen soll mit Hilfe von Propensity Score Matching erfolgen. Bisherige Ergebnisse zeigen, dass das UP für die Angststörungen so effektiv wie störungsspezifische Therapie ist (Barlow et al., 2017) und dass transdiagnostische Onlinetherapie vergleichbare Effekte zu störungsspezifischer Onlinetherapie erzielt (Newby, Twomey, Yuan Li, & Andrews, 2016). Es wird deshalb davon ausgegangen, dass das transdiagnostische iUP der störungsspezifischen Online-Intervention TK-DepressionsCoach nicht unterlegen sein wird.

**Studie 3:** Wie erwähnt stehen Studien zur Effektivität des Unified Protocols an Personen mit Krankheitsängsten aus und das obwohl Krankheitsängste in der Allgemeinbevölkerung sehr verbreitet sind und ein eher unterversorgtes Störungsbild darstellen (Bleichhardt & Hiller, 2007). Ungeleitete Interventionen stellen einen Ansatzpunkt dar, um die Verfügbarkeit von Therapie weiter zu erhöhen. Bisher werden keine Hinweise zu Unterschieden zwischen einer geleiteten und ungeleiteten Online-Intervention für Krankheitsängste berichtet: Eine Studie von Hedman und Kollegen (2016) konnte zeigen, dass ungeleitete Behandlungsprogramme in der Therapie von Krankheitsängsten ähnlich wirksam sind wie geleitete Programme. Das Ziel der dritten Studie ist es deshalb, die Wirksamkeit des ungeleiteten iUPs für Menschen mit ausgeprägten Krankheitsängsten zu untersuchen. Dazu soll das iUP mit einem randomisiert-kontrollierten Design mit einer aktiven Kontrollgruppe, die ein ungeleitetes Online Achtsamkeitstrainings absolviert, verglichen werden. Die Förderung von Achtsamkeit hat in den letzten Jahren einen großen Stellenwert in kognitiv verhaltenstherapeutischen Ansätzen eingenommen. Meta-Analysen bestätigen die Effektivität von Achtsamkeitsprogrammen für eine Bandbreite von klinischen und nichtklinischen Stichproben (Baer, 2003; Grossman, Niemann, Schmidt, & Walach, 2004; Hofmann, Sawyer, Witt, & Oh, 2010). Auch im Bereich der Krankheitsängste belegen mehrere Studien die Wirksamkeit achtsamkeitsbasierter Ansätze (Lovas & Barsky, 2010; Luberto, Magidson, & Blashill, 2017; McManus, Surawy, Muse, Vazquez-Montes, & Williams, 2012). Für die Angststörungen gibt es erste Hinweise, dass auch eine internetbasierte Förderung der Achtsamkeit zu einer Reduktion von Stress, Angst- und Depressionssymptomen führt (Boettcher et al., 2014). Studien zu den Effekten internetbasierter Achtsamkeitsprogramme für die Krankheitsängste stehen bisher aus.

## Methoden und Studiendesign

### Rekrutierung und Selektion der Teilnehmer*innen

**Studie 1-3**: Die Rekrutierung der Teilnehmer*innen erfolgt über Ankündigungen in Internet-Gesundheitsforen. Potenzielle Teilnehmer*innen werden auf einer Studienwebsite über Ziele, Ablauf sowie mögliche Nutzen und Risiken der geplanten Studie informiert (siehe Anhang A, exemplarisches Informationsschreiben für die Pilotstudie). Wenn die Teilnehmer*innen an einer Teilnahme interessiert sind, werden sie gebeten, die Einwilligungserklärung auszufüllen, zu unterschreiben und postalisch an die Studienleitung zu schicken (siehe Anhang B, exemplarische Einwilligungserklärung für die Pilotstudie). Die Teilnahme an der Studie ist freiwillig. Es gibt jederzeit die Möglichkeit, die Teilnahme zu jedem Zeitpunkt ohne Nennung von Gründen zu unterbrechen oder abzubrechen. Nachdem die Einwilligungserklärung bei der Studienleitung eingegangen ist, werden Teilnehmer per E-Mail eingeladen, sich mit ihrer E-Mail-Adresse, einem selbstgewählten Benutzernamen und einem Passwort zu registrieren.

**Studie 1 und 2:** Der Einschluss der Teilnehmer*innen erfolgt zweistufig: In einem ersten Schritt füllen Patienten demografische, störungsübergreifende und störungsspezifische Fragebögen aus (siehe Erhebungsinstrumente). Wenn die Teilnehmer*innen zu diesem Zeitpunkt über dem Cut-Off von mindestens einem störungsspezifischen Fragebogen liegen, werden sie per E-Mail kontaktiert, um ein Telefoninterview zu vereinbaren.

Im Telefoninterview wird mit dem Strukturierten Klinischen Interview für DSM-IV eine oder mehrere Diagnosen ermittelt (Wittchen, Wunderlich, Gruschwitz, & Zaudig, 1997). Bei den Interviewer*innen handelt es sich um die zwei Antragstellerinnen und um Honorarkräfte, die Master-Studierende der Klinischen Psychologie sind und die in der Durchführung des SKIDs trainiert wurden. Alle Interviewer unterliegen der Schweigepflicht.

**Studie 3:** Nach Ihrer Einwilligung füllen Patienten demografische, störungsübergreifende und störungsspezifische Fragebögen aus (siehe Erhebungsinstrumente). Wenn die Teilnehmer*innen auf der Subskala Krankheitsangst des Short Health Anxiety Inventory über dem Cut-Off liegen, werden sie in die Studie eingeschlossen. Dafür wird der Cut-Off auf 20 festgelegt, ein Cut-Off-Wert, der in früheren Studien verwendet wurde (Tyrer et al., 2017) und in der deutschen Validierungsstudie eine gute Balance aus Spezifität und Sensitivität zeigte (Bailer et al., 2013).

Stichprobengröße

**Studie 1:** Ausgehend von einem zu erwartenden großen Effekt (Cohen’s *d* = .8) zugunsten der Interventionsgruppe (einseitiger t-Test für unabhängige Stichproben, alpha=0.05) und einer Power von 80%, zielen wir auf eine Stichprobe von *N*=42 Teilnehmenden ab. Bei einem erwarteten Drop-out von maximal 15% zur Post-Erhebung ergibt sich daraus eine zu rekrutierende Stichprobe von *N*=60.

**Studie 2:** Die Studie untersucht die Nichtunterlegenheit des iUP im Vergleich zu einer depressionsspezifischen Onlineintervention. Es wird empfohlen, den Non-Inferiority Margin anhand von historischen Ergebnissen zum Vergleich der Kontrollintervention und Placebo festzulegen, häufig wird er dabei auf eine Größe, die 50% der in diesen Studien beobachteten Effektstärke entspricht, festgelegt (Althunian, Boer, Groenwold, & Klungel, 2017; European Medicines Agency Committee For Medicinal Products For Human Use [CHMP]). Meta-Analysen zeigen für begleitete Online-Interventionen für depressive Störungen eine gepoolte Effektstärke von *d*=.78 (Richards & Richardson, 2012). Entsprechend wird der Non-Inferiority Margin Δ auf 0,39 (.78x0.5) festgelegt. Übersetzt auf das primäre Outcome-Maß PHQ-9 bedeutet dieser Margin, dass die untere Grenze des 95% Konfidenzintervalls um den mittleren Unterschied zwischen den Behandlungen -2,7 PHQ Punkte nicht überschreiten darf (angenommene SD des Differenzwerts = 6.9 (Saxon et al., 2017)). Um dies zu zeigen, ist bei einer Power von 95% und alpha=0.05 eine Stichprobe von *N*=143 nötig. Bei einem erwarteten Drop-out von maximal 15% zur Post-Erhebung ergibt sich daraus eine zu rekrutierende Stichprobe von *N*=168.

**Studie 3**: Ausgehend von einem kleinen bis mittleren zu erwartenden Effekt (Cohen’s *d* =.4) zugunsten des iUP-Programms (einseitiger t-Test für unabhängige Stichproben, alpha = 0.05) und einer Power von 80% zielen wir auf eine Stichprobe von *N*=156 Teilnehmenden ab. Bei einem erwarteten Drop-out von maximal 15% zur Post-Erhebung ergibt sich daraus eine zu rekrutierende Stichprobe von *N*=184.

Ein- und Ausschlusskriterien

Teilnehmer*innen werden eingeschlossen, wenn sie **a)** mindestens 18 Jahre alt sind, **b)** Zugang zum Internet haben, **c)** an einem Telefoninterview teilnehmen können, **d)** für **Studie 1:** eine mit dem SKID gestellte Primärdiagnose von Generalisierter Angststörung, Sozialer Angststörung, Panikstörung, Agoraphobie, Depression (einzelne Episode oder rezidivierend), Dysthymia, Somatischer Belastungsstörung mit vorwiegenden Krankheitsängsten oder eine Krankheitsangststörung haben, für **Studie 2**: eine mit dem SKID gestellte Primärdiagnose der Depression (einzelne Episode oder rezidivierend) oder Dysthymia haben und für **Studie 3**: eine erhöhte Krankheitsangst, definiert als Score über 20 auf der Krankheitsangstsubskala des Short Health Anxiety Inventory , aufweisen, **e)** sich nicht bereits in anderer psychotherapeutischer Behandlung oder Beratung befinden, **f)** seit drei Monaten auf einer stabilen Medikamentendosis eingestellt sind, sollten sie Medikamente für die Ängste oder Depression einnehmen, **g)** keine akuten psychotischen Symptome oder Substanzabhängigkeit aufweisen und **h)** nicht akut suizidgefährdet sind.

Suizidgedanken werden mit dem Suiziditem des PHQ-9 (Kroenke & Spitzer, 2002; Löwe, Kroenke, Herzog, & Gräfe, 2004) erhoben. Beantworten Teilnehmer*innen im Rahmen des Screenings dieses Item mit >1, wird ihr Suizidrisiko im Rahmen des diagnostischen Interviews evaluiert. Interviews mit potentiell suizidalen Teilnehmer*innen werden nicht von Masterstudierenden sondern von einer der zwei Antragsstellerinnen durchgeführt. Die erste Antragstellerin ist approbierte Verhaltenstherapeutin und die zweite Antragstellerin befindet sich in fortgeschrittener Weiterbildung und verfügt über die Behandlungserlaubnis KVT. Teilnehmer*innen mit akutem Suizidrisiko werden von der Studie ausgeschlossen und an lokale Psychiater*innen oder Psychotherapeut*innen verwiesen.

### Aufwandsentschädigung

Die Teilnehmer*innen aller drei Studien werden für Ihre Teilnahme an der onlinebasierten Intervention nicht entschädigt.

### Studiendesign

**Studie 1:** Die geplante Studie folgt einem randomisiert-kontrollierten Design. Die Teilnehmer*innen werden zufällig der Interventionsgruppe oder der Wartegruppe zugeteilt. Die Randomisierung erfolgt per Zufallszahlen, unabhängig von der Untersuchungsleiterin. Nach dem Telefoninterview erhalten die Teilnehmer*innen der Interventionsgruppe per E-Mail Zugang zu der onlinebasierten Intervention. Teilnehmer*innen der Wartegruppe erhalten nach zehn Wochen Wartezeit ebenfalls Zugang zum iUP.

**Studie 2:** Die Studie ist eine nicht-randomisierte Nichtunterlegenheitsstudie. Teilnehmer*innen am iUP werden zu einer bestehenden Stichprobe von N>2000 Teilnehmenden des TK-DepressionsCoach gematcht, um auf diese Weise eine Kontrollgruppe zu konstruieren. Für alle Teilnehmer*innen wird ein Propensity Score – die geschätzte Wahrscheinlichkeit, in Abhängigkeit von vorher definierten möglichen Kovariablen in der Gruppe zu sein – bestimmt. Kovariablen anhand derer der Propensity Score geschätzt werden soll sind z.B. Schweregrad der Depression, komorbide Störungen, vorherige psychotherapeutische Behandlung, Medikamenteneinnahme, Alter, Geschlecht, Schulbildung und Beziehungsstatus. Dieses Vorgehen erlaubt es, für jeden Teilnehmer der iUP-Gruppe basierend auf dem Propensity Score genau einen „nearest neighbour“ aus der TK-DepressionsCoach-Stichprobe zu bestimmen und auf diese Weise eine möglichst vergleichbare und gleich große Kontrollgruppe zu schaffen. Die Veränderung der depressiven Symptomatik wird dann zwischen dem störungsspezifischen TK-DepressionsCoach und dem transdiagnostischen iUP verglichen.

**Studie 3:** Die Studie folgt einem randomisiert-kontrollierten Design mit einer aktiven Kontrollgruppe. Die aktive Kontrollgruppe wird über den Verlauf der 10 Wochen ein unbegleitetes Achtsamkeitsprogramm absolvieren, dessen Achtsamkeitsübungen auf der Mindfulness-Based Stress Reduction (Kabat-Zinn, 1982) basieren. Teilnehmer*innen in der Achtsamkeitsgruppe werden wöchentlich eine Einführung erhalten und dann selbstständig mit Hilfe von bereitgestellten Audiodateien eine achtsame Haltung üben. Die Teilnehmer werden angehalten, ihre Erfahrung und ihre Fortschritte innerhalb des Programmes zu protokollieren.

### Intervention - iUP

Die Nutzung der onlinebasierten Intervention ist auf 10 Wochen angelegt. Die Module befassen sich mit Motivation und Zielen (Modul 1), Emotionen (Modul 2), Achtsamkeit (Modul 3), kognitiver Flexibilität (Modul 4 und 5), Emotionsvermeidung (Modul 6), der Toleranz körperlicher Empfindungen (Modul 7), der Konfrontation mit Situationen oder Gedanken, die starke Emotionen auslösen und die Teilnehmer*innen bisher vermieden haben (Modul 8 und 9) und der Rückfallprophylaxe (Modul 10). In jedem Modul finden die Teilnehmer*innen psychoedukative Inhalte und Übungen, welche sie protokollieren. Wir empfehlen den Teilnehmer*innen pro Woche mindestens 60 Minuten für die Beschäftigung mit den Inhalte einzuplanen, die Teilnehmer können allerdings selbst entscheiden wie viel Zeit sie investieren und wie sie die Bearbeitung einteilen. In der geleiteten Version des iUPs ist ein regelmäßiger Kontakt zu den Berater*innen vorgesehen ist. Die Teilnehmer*innen erhalten, nachdem sie eine Woche lang an einem Modul gearbeitet haben, personalisiertes Feedback auf ihre Arbeit und erhalten Zugang zum nächsten Modul. In der ungeleiteten Version des iUPs erhalten Teilnehmer*innen automatisiertes Feedback und erhalten im Anschluss Zugang zum nächsten Modul. In beiden Versionen erhalten die Teilnehmer*innen zu Beginn der Intervention den Hinweis, dass sie sich per Nachricht melden können, wenn Fragen oder Schwierigkeiten auftreten.

**Online-Berater*innen:** Online-Berater*innen sind die Antragstellerin Carmen Schäuffele sowie Studierende der Klinischen Psychologie, die in dem Projekt ihre Masterarbeit schreiben. Beide Antragsstellerinnen haben an der Boston University ein Training im Unified Protocol absolviert. Masterstudierende erhalten eine Einführung in das Programm und üben dann an Muster-Patienten das Schreiben des Online-Feedbacks. Für den ersten Behandlungsfall wird das entworfene Feedback von einer der zwei Antragsstellerinnen geprüft und gegebenenfalls korrigiert. Über den Zeitraum der Studie finden wöchentlich Supervisionssitzungen mit der ersten Antragstellerin statt.

### Erhebungsinstrumente

Klinisches Interview: Ein Telefoninterview mit dem SKID (Wittchen et al., 1997) wird in Studie 1 und 2 zu zwei Zeitpunkten durchgeführt: Vor der Behandlung zur Diagnosestellung und nach der Behandlung zur Neubewertung der gestellten Diagnosen.

Die Teilnehmer*innen werden zu Beginn eines jeden neuen Moduls einen kurzen Fragebogen und eine umfangreichere Messbatterie vor, nach der Hälfte und nach der Behandlung ausfüllen. Eine Follow-up-Erhebung wird nach 3, 6 und 12 Monaten stattfinden. Außerdem werden die Teilnehmer*innen nach einigen Modulen Prozessmaße zu Fertigkeiten, die in diesem Modul erlernt werden sollen, ausfüllen

Für eine wöchentliche Veränderungsmessung wird der Patientengesundheitsfragebogen PHQ-4 (Kroenke, Spitzer, Williams, & Löwe, 2009; Löwe et al., 2010) eingesetzt. Für Studie 1 und 2, in die auch Depressive eingeschlossen werden, soll außerdem ein Item, das Suizidalität erfragt, eingesetzt werden, um Hinweise auf eine mögliche Suizidalität zu geben. Teilnehmer*innen, die in einer der wöchentlichen Erhebungen verstärkte Suizidgedanken angeben (Suiziditem>1) werden telefonisch von einer der Antragstellerinnen kontaktiert.

Als primäres Maß wird in Studie 1 der General Health Questionnaire GHQ-12 (Goldberg et al.; Schmitz, Kruse, & Tress, 1999), in Studie 2 der Patientengesundheitsfragebogen PHQ-9 (Kroenke & Spitzer, 2002; Löwe et al., 2004) und in Studie 3 die Kurzform des Health Anxiety Inventory eingesetzt (Bailer et al., 2013; Salkovskis, Rimes, Warwick, & Clarke, 2002).

Zu den weiteren diagnosespezifischen Fragebögen gehören: der Patientengesundheitsfragebogen PHQ-9 für Depressionen (Kroenke & Spitzer, 2002; Löwe et al., 2004), die Liebowitz-Soziale Angst-Skala für die Soziale Angststörung (Heimberg et al., 1999; Stangier & Heidenreich, 2005), die Kurzform des Health Anxiety Inventory für Somatische Belastungsstörung und Krankheitsangststörung (Bailer et al., 2013; Salkovskis et al., 2002), die Panik- und Agoraphobie-Skala für die Panikstörung und Agoraphobie (Bandelow, 2016), der Generalized Anxiety Disorder Screener GAD-9 für die Generalisierte Angststörung (Löwe et al., 2008) und der Short-Form Health Survey SF-12 für gesundheitsbezogene Lebensqualität (Bullinger & Kirchberger, 2011; Ware Jr, Kosinski, & Keller, 1996). Außerdem werden Beeinträchtigungen im Zusammenhang mit somatischen Symptomen mit einer visuellen Analogskala bewertet.

Zu den Prozessmaßnahmen gehört der Southhampton Mindfulness Questionnaire SMQ für Achtsamkeit (Chadwick et al.., 2008), die Subskala zur Neubewertung des Emotion Regulation Questionnaire ERQ für kognitive Flexibilität (Gross & John, 2003; Abler & Kessler, 2009), die Brief Experiential Avoidance Scale BEAQ für Emotionsvermeidung (Gámez et al.., 2014) und die Behavioral Activation Depression Scale zur Vermeidung auf der Verhaltensebene (Fuhr et al., 2016; Manos, Kanter, & Luo, 2011).

Um negative Auswirkungen der Behandlungen zu beurteilen, wird der Negative Effects Questionnaire NEQ nach der Behandlung ausgefüllt (Rozental, Kottorp, Boettcher, Andersson, & Carlbring, 2016).

## Risiken und mögliche Folgeeffekte für die Teilnehmer*innen

In den geplanten Studien werden keine Blut- oder Gewebeproben entnommen. Es findet keine Prüfung eines Arzneimittels statt. Es liegt keine Täuschung von Studienteilnehmer*innen vor.

Die Teilnahme an der iUP Intervention ist mit einem voraussichtlichen Nutzen verknüpft. Studien weisen darauf hin, dass internetbasierte Interventionen sowohl geleitet als auch ungeleitet hilfreich in der Reduktion ängstlicher und depressiver Symptome sein können (siehe Einleitung). Für das Unified Protocol gibt es außerdem für die face-to-face-Behandlung Hinweise, dass die Behandlung der Angststörungen zu einer Reduktion der Angstsymptomatik (Barlow et al., 2017; Farchione et al., 2012) und zu einer gesteigerten Lebensqualität führt (Gallagher et al., 2013). Für depressive und somatoforme Störungen stehen randomisiert kontrollierte Studien allerdings aus.

Das potenzielle Risiko einer Teilnahme am iUP ist aufgrund bisheriger empirischer Befunde als gering einzuschätzen. Circa 5% der Teilnehmer*innen in ähnlichen Online-Therapie Programmen berichten, dass sie einen Anstieg von Stress, Angst oder anderen unangenehmen Gefühlen erleben. In den allermeisten Fällen sind diese nur temporär und haben keine negativen Folgen (Rozental, Magnusson, Boettcher, Andersson, & Carlbring, 2017).

Es ist aufgrund der Ergebnisse bisheriger Studien anzunehmen, dass auch die Teilnahme an einer unbegleiteten Achtsamkeitsintervention (aktive Kontrollgruppe Studie 3) zu einer Verbesserung des allgemeinen Wohlbefindens und einer Reduktion der Ängste und depressiven Symptomatik führt (Boettcher et al., 2014; Spijkerman, Pots, & Bohlmeijer, 2016). Bisherige Befunde zeigen außerdem, dass Achtsamkeitsübungen trotz der Fokussierung der Aufmerksamkeit auf körperliche Vorgänge nicht zu einer Verschlechterung der Krankheitsängste führen (McManus et al., 2012; Williams, McManus, Muse, & Williams, 2011).

Die Teilnehmer*innen werden über voraussichtlichen Nutzen und potentielles Risiko detailliert aufgeklärt (siehe Anhang A).

1. Umgang mit Zufallsbefunden

Für Studie 1 und 2 werden Teilnehmer*innen aufgrund der Screening-Fragebögen und des klinischen diagnostischen Interviews in die Studie eingeschlossen und für Studie 3 aufgrund eines Wertes über dem Cut-Off auf dem Short Health Anxiety Inventory.

Auf Grund des Einschlusses depressiver Patienten werden im Rahmen der Studie auch Instrumente eingesetzt, die Items zu Suizidalität enthalten. Patienten werden im Rahmen der Studie über den Umgang mit möglichen suizidalen Krisen aufgeklärt und erhalten Anlaufstellen und Telefonnummern. Geben die Teilnehmer*innen im Screening oder den Erhebungen innerhalb der Intervention an, unter Suizidgedanken zu leiden, werden sie von den Versuchsleiterinnen telefonisch kontaktiert.

Die Ergebnisse des SKID-Interviews werden den Patient*innen auf Grund des Ferndiagnoseverbotes nicht als Diagnose mitgeteilt. Eine solche Ferndiagnose würde nicht durch eine somatische Abklärung gestützt werden und die rechtlichen Voraussetzungen der persönlichen face-to-face Aufklärung nicht erfüllen.

1. Vorkehrungen zum Datenschutz

Die Erhebung, Speicherung und Auswertung der Studiendaten unterliegt den Bestimmungen des Berliner Datenschutzgesetzes. Folgende Maßnahmen werden zu den unterschiedlichen Studienphasen ergriffen, um die Sicherheit der Daten zu gewährleisten.

**6.1. Erhebung**

Die erhobenen Daten werden zunächst pseudonymisiert. Personenbezogene Angaben werden nur soweit erhoben, wie sie für die Durchführung der Studie notwendig sind. Vor der Registrierung werden die Teilnehmer*innen gebeten, die Einwilligungserklärung (siehe Anhang B) auszudrucken, zu unterschreiben und postalisch an die Studienleitung zu schicken. Auf der Einverständniserklärung werden Name, Adresse, Telefonnummer, und E-Mail-Adresse angegeben. Die Einwilligungserklärungen werden in einem verschlossenen Schrank zehn Jahre lang aufbewahrt.

Online werden allgemeine demographische Angaben (Alter, Geschlecht, Familienstand, Schulbildung, aktuelle und vorherige psychotherapeutische Behandlung und Medikamenteneinnahme) erfasst sowie Pseudonym/Nutzername und Passwort zur Registrierung. Außerdem wird eine E-Mail-Adresse erfragt. Diese dient dem Zwecke der Passwortzurücksetzung, der Erinnerung sich regelmäßig einzuloggen und der Kontaktaufnahme zu den Follow-Up-Zeitpunkten. Die Profildaten werden ausschließlich innerhalb einer passwortgeschützten Onlineplattform abgefragt und gespeichert (unter Verwendung einer SSL-gesicherten Datenübertragung) und nach der letzten Erhebung gelöscht.

Teilnehmer*innen können so lange die Löschung ihrer Daten verlangen wie der Datensatz ihrer Person zugeordnet werden kann. Im Anschluss an die Analyse werden die Daten in anonymisierter Form archiviert und ausschließlich für wissenschaftliche Zwecke verwendet.

**6.2. Speicherung**

Die Screeningdaten der ersten Fragebogenbefragung werden auf derselben Plattform wie das Onlineprogramm erhoben und unter Beachtung der gesetzlichen Bestimmungen und unter Wahrung etablierter Sicherheitsstandards gespeichert.

Die im Rahmen des Programms anfallenden Daten („Nutzungsdaten“, wie z.B. Häufigkeit der Logins) werden pseudonymisiert gespeichert. Die Sicherheit des Internetservers wird kontinuierlich durch einen Sicherheitsscan kontrolliert. Es werden keinerlei weitere Informationen der Teilnehmenden (z.B. Bewegungsprofile, Zugriff auf Internetseiten) gespeichert. Die Daten werden über SSL-Verschlüsselung an einen Server des deutschen Serveranbieters Hetzner (Hetzner Online GmbH) versendet, deren Server nach ISO/IEC 27001 zertifiziert sind und damit hohe europäischen Datenschutz-Standards erfüllen.

Die vollständig anonymisierten Daten werden nach dem Abschluss der Studien über die Internet-Datenbank Open Science Framework öffentlich zugänglich gemacht. Dieses Vorgehen dient der Sicherstellung guter wissenschaftlicher Arbeit.

**6.3. Auswertung**

Der Abruf der gespeicherten pseudonymisierten Daten zu Forschungs-/Auswertungszwecken ist passwortgeschützt. Das Passwort ist nur den Projektleiterinnen des zur Begutachtung vorliegenden Forschungsprojektes zugänglich. Es verpflichten sich alle Projektmitarbeiterinnen schriftlich zur Verschwiegenheit über personenbezogene Daten und zur Einhaltung des Berliner Datenschutzgesetzes.

Es werden keine Auswertungen und Berichte erstellt, die Rückschlüsse auf einzelne Personen ermöglichen. Kleine Personengruppen (z.B. Altersgruppen) werden nicht gesondert dargestellt.

## Finanzierung der Studie

Die Studie wird aus Haushaltsmitteln finanziert. Es liegt keine Aufforderung eines Drittmittelgebers zur ethischen Begutachtung vor.

## Erklärung

Mir ist die Ordnung zur ethischen Begutachtung von Forschungsprojekten des Fachbereichs Erziehungswissenschaft und Psychologie der Freien Universität Berlin bekannt. Mir ist bekannt, dass ich gemäß §19 Berliner Datenschutzgesetz (BlnDSG) verpflichtet bin, für automatisierte Verarbeitungen personenbezogener und personenbeziehbarer Daten eine Datei- und Verfahrensbeschreibung zu erstellen und diese gemäß §19a dem behördlichen Datenschutzbeauftragten der FU Berlin zur Verfügung stellen muss.

Ein Antrag zur ethischen Begutachtung des Forschungsprojektes „Machbarkeit, Akzeptanz und Wirksamkeit der Online-Intervention iUP“ ist bisher an keiner anderen Stelle eingereicht.

Johanna Böttcher Carmen Schäuffele

Berlin, März 2018

**Anhang A:** Exemplarische Teilnehmerinformation für die Teilnehmer*innen der Pilotstudie

| **Informationsblatt vom 14.03.2018 zur Studie  „iUP – eine onlinebasiertes Programm für Menschen mit Ängsten und Depressionen “** | |
| --- | --- |
| Ort der Studie | Freie Universität Berlin  Arbeitsbereich Klinische Psychologie und Psychotherapie  Habelschwerdter Allee 45, D-14195 Berlin |
|  |  |
| Verantwortliche | Dr. Johanna Böttcher (FU Berlin) johanna.boettcher@fu-berlin.de |

**Liebe Interessentin, lieber Interessent,**

wir freuen uns, dass Sie erwägen, im Rahmen unserer Studie das Onlineprogramm „iUP“ auszuprobieren. Wir untersuchen iUP im Rahmen einer wissenschaftlichen Studie, die von der Freien Universität Berlin durchgeführt wird. Im Folgenden möchten wir Sie über die Ziele und den Ablauf der Studie sowie über den Datenschutz aufklären.

**Hintergrund**

Wir alle kennen Ängste, machen uns Sorgen oder sind mal niedergeschlagen. Das ist völlig normal! Wenn diese Ängste, Sorgen oder Niedergeschlagenheit jedoch sehr stark ausgeprägt sind und ein starker Leidensdruck entsteht, sprechen Psychologen von einer psychischen Störung. Weil bei diesen Störungen intensive Gefühle wie Angst oder Traurigkeit im Vordergrund stehen, nennt man diese Störungen auch „emotionale Störungen“. Zu den emotionalen Störungen zählen z.B. die Angststörungen oder depressiven Störungen.

Das Onlineprogramm iUP, das wir in dieser Studie untersuchen möchten, setzt genau da an. Das iUP basiert auf dem Behandlungskonzept Unified Protocol und wurde ursprünglich an der Universität Boston entwickelt. Das Besondere an dem Programm ist, dass alle emotionalen Störungen, d.h. alle Störungen bei denen Gefühle eine Rolle spielen, damit behandelt werden können – auch wenn jemand mehr als eine solche emotionale Störung hat.

**Ziele**

Ziel dieser Studie ist es, die Machbarkeit und Wirksamkeit von iUP für Menschen mit emotionalen Störungen zu überprüfen. Erste Untersuchungen in den USA haben bereits gezeigt, dass das Unified Protocol Menschen mit solchen Beschwerden helfen kann. In dieser Studie soll nun untersucht werden, ob das Unified Protocol auch wirksam ist, wenn es als Onlineprogramm eingesetzt wird, d.h. wenn die gesamte Therapie über das Internet erfolgt (siehe unten).

Um die Wirkung von iUP zu überprüfen, müssen die Nutzer des Programms (Behandlungsgruppe) mit einer Gruppe von Personen verglichen werden, die in der selben Zeit das Programm nicht nutzen und auch sonst keine andere Behandlung für Ihre psychischen Beschwerden wahrnehmen (Wartekontrollgruppe). Diese Personen befindet sich sozusagen auf einer Warteliste - nach Ende des Nutzungszeitraums (10 Wochen) der Behandlungsgruppe, erhält die Wartekontrollgruppe die Möglichkeit, das Programm im selben Umfang zu nutzen wie die Behandlungsgruppe vor ihr. Der Vergleich einer aktiven Behandlungsgruppe mit einer Wartekontrollgruppe ist ein notwendiger Standard in der Überprüfung psychologischer Programme.

Mit einer Teilnahme an unserer Studie würden Sie sich bereit erklären, einer der beiden Nutzergruppen zugelost zu werden: entweder der Behandlungsgruppe, die mit der aktiven Nutzung des Programms sofort beginnt, oder der Kontrollgruppe, die nach einer Wartezeit von 10 Wochen mit dem Programm beginnt.

**Ablauf**

Lassen Sie uns nun den Ablauf der Studie erklären:

Am Anfang möchten wir herausfinden, ob das angebotene Programm das Richtige für Sie ist. Dieser Abklärungsprozess ist wichtig, weil unser Ansatz nur für Personen geeignet ist, die unter einer emotionalen Störung leiden. Der Abklärungsprozess läuft folgendermaßen:

Nachdem Sie der Teilnahme an der Studie zugestimmt haben und uns die Einwilligungserklärung per Post zugeschickt haben, können Sie sich registrieren. Anschließend füllen Sie online einige Fragebögen zu Verhalten und Gefühlen aus. Weisen die Fragebögen darauf hin, dass bei Ihnen eine emotionale Störung vorliegen könnte, werden wir mit Ihnen einen Termin für ein Telefoninterview vereinbaren. In diesem Telefoninterview werden wir Ihnen anhand eines strukturierten Leitfadens Fragen stellen um sicherzugehen, dass die Teilnahme an dem Programm genau das Richtige für Sie ist. Wenn auch die Ergebnisse des Telefoninterviews darauf hindeuten, dass wir Sie mit dem iUP gut bei Ihren Beschwerden unterstützen können, werden Sie per Zufall der Behandlungsgruppe oder der Wartekontrollgruppe zugeteilt. Landen Sie in der Behandlungsgruppe schalten wir Sie sofort für das Programm frei. Ein Online-Berater wird Ihnen zugeteilt. In der Wartekontrollgruppe melden wir uns nach 10 Wochen wieder und laden Sie zur Programmteilnahme ein.

Dann kann es mit der Behandlung losgehen. Insgesamt ist das Programm auf 10 Wochen angelegt, wobei Sie selbst bestimmen können, wie häufig oder intensiv Sie die Übungen absolvieren möchten. In den Übungen erlernen Sie Fertigkeiten zum Umgang mit überwältigenden Gefühlen. Dies erfordert viel Übung und eine längere Beschäftigung mit den Inhalten. Wir empfehlen Ihnen daher mindestens 60 Minuten pro Woche mit dem Programm zu arbeiten. Einmal in der Woche erhalten Sie Feedback von Ihrem persönlichen Berater. Wir werden Ihnen auch regelmäßig Fragen zu Ihren aktuellen Beschwerden stellen, um auch kurzfristige Entwicklungen verfolgen zu können. Ihre Fortschritte werden in pseudonymisierter Form automatisch gespeichert.

Damit wir auswerten können, ob das Programm wirkt, werden wir Sie bitten, wöchentlich einen kurzen Fragebogen und die Fragebögen vom Anfang nach der Hälfte und nach dem Programm noch einmal ausfüllen. Außerdem werden wir 3, 6 und 12 Monate nach Abschluss des Programmes noch einmal per E-Mail Kontakt zu Ihnen aufnehmen und Sie bitten, wiederum die Fragebögen auszufüllen.

Nach der Programmbeendigung werden wir Sie außerdem bitten, an einem erneuten Telefoninterview teilzunehmen. Hier möchten wir überprüfen, wie sich Ihre Beschwerden entwickelt haben.

**Freiwilligkeit und Anonymität**

Die Teilnahme an der Studie ist freiwillig. Sie können jederzeit und ohne Angabe von Gründen die Teilnahme an dieser Studie beenden, ohne dass Ihnen daraus Nachteile entstehen.

Die im Rahmen dieser Studie erhobenen Daten und persönlichen Mitteilungen werden vertraulich behandelt. So unterliegen alle Projektmitarbeiter, die durch direkten Kontakt mit Ihnen über personenbezogene Daten verfügen, der Schweigepflicht. Des Weiteren wird die Veröffentlichung der Ergebnisse der Studie in anonymisierter Form erfolgen, d. h. ohne dass Ihre Daten Ihrer Person zugeordnet werden können.

**Datenschutz**

Im Rahmen der Studie erheben wir verschiede persönliche Daten von Ihnen. Zum einen erheben wir mit der Einwilligungserklärung Ihren Namen, Ihre Anschrift, Ihre Telefonnummer und E-Mail-Adresse. Dann erheben wir die Daten aus dem Telefoninterview sowie die Daten aus den Fragebögen, die Sie zu unterschiedlichen Zeiten während der Studie und im Anschluss an die Beendigung der Studie am Computer ausfüllen. Außerdem wird Ihr persönlich gewählter Benutzername, Ihr Passwort sowie eine E-Mail-Adresse gespeichert. Schließlich erheben wir Daten zur Nutzung des Programms, die automatisch gespeichert werden, z.B. wie häufig Sie sich in das Programm einloggen.

Wir haben folgende Maßnahmen getroffen, um die Vertraulichkeit Ihrer Daten zu gewährleisten:

1) Die postalische Einwilligungserklärung wird separat von den anderen Daten in einem verschlossenen Schrank aufbewahrt.

2) Die Übermittlung Ihrer Daten via Internet erfolgt ausschließlich verschlüsselt. Dabei

kommt eine Technik zum Einsatz, die zum Beispiel auch Banken im internetbasierten

Zahlungsverkehr einsetzen (SSL-Verschlüsselung).

3) Alle digitalen Daten werden pseudonymisiert ohne Angabe Ihres Namens gespeichert. Nach der letzten Datenerhebung werden die Daten dann anonymisiert. Damit ist es niemandem mehr möglich, die erhobenen Daten mit Ihrem Namen in Verbindung zu bringen. Bis zur letzten Erhebung, können Sie die Löschung aller von Ihnen erhobenen Daten verlangen. Ist der Datensatz erst einmal anonymisiert, können wir Ihren Datensatz nicht mehr identifizieren. Deshalb können wir Ihrem Wunsch nach Löschung Ihrer Daten nur bis zu der letzten Erhebung nachkommen.

3) Das Programm wird auf einem externen Server des deutschen Serveranbieters Hetzner (Hetzner Online GmbH) gehostet, der nach ISO/IEC 27011 zertifiziert ist und somit höchsten Sicherheitsstandards entspricht

.4) Alle im Rahmen dieser Studie erhobenen Daten und persönlichen Mitteilungen unterliegen

der Schweigepflicht.

Die vollständig anonymisierten Daten werden über die Internet-Datenbank Open Science Framework öffentlich zugänglich gemacht. Dieses Vorgehen dient der Sicherstellung guter wissenschaftlicher Arbeit. Andere Forschende können dadurch beispielsweise die Auswertung nachvollziehen oder eine alternative Auswertung testen.

**Nutzen und Risiken bei einer Teilnahme an der Studie**

Die Studienteilnahme ist mit einem voraussichtlichen Nutzen verbunden, dem andererseits

ein gewisser Aufwand sowie mögliche Risiken gegenüberstehen.

*Möglicher Nutzen:* Die Teilnahme am iUP-Programm ist mit einem voraussichtlichen Nutzen verknüpft. Studien weisen darauf hin, dass internetbasierte Behandlungsprogramme hilfreich in der Reduktion ängstlicher und depressiver Symptome sein können. Mit iUP können Sie Fertigkeiten zum Umgang mit Gefühlen trainieren, von denen man weiß, dass sie mit einer Erhöhung psychischer Gesundheit und Wohlbefinden verknüpft sind. Da das in dieser Studie realisierte onlinebasierte Programm noch nicht untersucht wurde, handelt es sich bei den erwarteten positiven Effekten um Hypothesen, die gegebenenfalls nach Studienabschluss durch wissenschaftliche Resultate untermauert werden können.

*Mögliche Risiken:* Ähnlich wie medikamentöse Behandlungen können auch psychologische Programme Nebenwirkungen haben. Ungefähr 5% der Teilnehmer*innen in ähnlichen Programmen berichten, dass sie einen Anstieg von Stress, Angst oder anderen unangenehmen Gefühlen erleben. In den allermeisten Fällen sind diese nur temporär und haben keine negativen Folgen. Trotzdem wollen wir Sie ermutigen, uns oder Ihren Online-Berater direkt anzusprechen, wenn Sie eine Verschlechterung Ihrer Beschwerden spüren. Wir möchten Sie außerdem darauf hinweisen, dass die Benutzung des Onlineprogramms eine psychologische, psychiatrische oder medizinische Behandlung nicht ersetzen kann.

Die Teilnahme an der Studie und die Nutzung von iUP ist kostenlos und wird nicht vergütet.

**Ethikkommission**

Die Studie wurde durch die Ethikkommission des Fachbereichs Erziehungswissenschaften und Psychologie der Freien Universität Berlin genehmigt.

**Anhang B:** Exemplarische Postalische Einwilligungserklärung für die Pilotstudie

| **Einwilligungserklärung zur Studie  „iUP – eine onlinebasierte Behandlung für Menschen mit Ängsten und Depressionen “** | |
| --- | --- |
| Ort der Studie | Freie Universität Berlin  Arbeitsbereich Klinische Psychologie und Psychotherapie  Habelschwerdter Allee 45, 14195 Berlin |
|  |  |
| Verantwortliche | Dr. Johanna Böttcher (FU Berlin) johanna.boettcher@fu-berlin.de |

| Name |  |
| --- | --- |
| Adresse |  |
| Telefonnummer |  |
| E-Mailadresse |  |

Bitte lesen Sie die folgenden Punkte sorgfältig durch!

Bitte fragen Sie nach, wenn Sie etwas nicht verstanden haben oder wissen möchten!

- - - 1. Ich habe die Informationen zur Pilotstudie, die auf der Studienwebsite abrufbar sind, sorgfältig gelesen und verstanden. Meine Fragen im Zusammenhang mit der Teilnahme an dieser Studie wurden zufriedenstellend beantwortet. Ich erhalte eine Kopie meiner unterschriebenen Einverständniserklärung.
      2. Ich weiß, dass die im Rahmen dieser Studie erhobenen Daten und persönlichen Mitteilungen der Schweigepflicht unterliegen. Ich bin einverstanden, dass an der Studie beteiligte Einsicht in meine Originaldaten nehmen dürfen, jedoch unter strikter Einhaltung der Vertraulichkeit. Während der ganzen Studie wird die Vertraulichkeit strikt gewahrt. Die Daten werden zunächst pseudonymisiert gespeichert und nach der letzten Befragung anonymisiert. Ich willige in die Datenverarbeitung meiner personenbezogenen Daten gemäß den Teilnehmerinformationen, die mir vorlagen, ein.
      3. Ich nehme an der Studie freiwillig teil. Ich kann jederzeit und ohne Angabe von Gründen meine Zustimmung zur Teilnahme widerrufen und bis zur letzten Befragung die Löschung meiner Daten verlangen, ohne dass mir deswegen Nachteile entstehen.

Ich bestätige durch meine Unterschrift, dass ich bereit bin, an der Studie mitzuwirken.

| Ort, Datum | Ihre Unterschrift |
| --- | --- |
| Ort, Datum | Unterschrift der an der Studie beteiligten Person (bitte freilassen)* |

Bitte senden Sie die ausgefüllte und unterschriebene Einwilligungserklärung an

**Dr. Johanna Böttcher, Freie Universität Berlin, Arbeitsbereich Klinische Psychologie und Psychotherapie, Habelschwerdter Allee 45, 14195 Berlin**

* Eine Kopie des von uns unterschriebenen Exemplars wird Ihnen postalisch zugesandt.

Literatur

Althunian, T. A., Boer, A. de, Groenwold, R. H. H., & Klungel, O. H. (2017). Defining the noninferiority margin and analysing noninferiority: An overview. *British journal of clinical pharmacology*, *83*(8), 1636–1642. https://doi.org/10.1111/bcp.13280

Andersson, G., & Cuijpers, P. (2009). Internet-based and other computerized psychological treatments for adult depression: A meta-analysis. *Cognitive behaviour therapy*, *38*(4), 196–205. https://doi.org/10.1080/16506070903318960

Andersson, G., & Titov, N. (2014). Advantages and limitations of Internet-based interventions for common mental disorders. *World psychiatry : official journal of the World Psychiatric Association (WPA)*, *13*(1), 4–11. https://doi.org/10.1002/wps.20083

Andrews, G., Cuijpers, P., Craske, M. G., McEvoy, P., & Titov, N. (2010). Computer therapy for the anxiety and depressive disorders is effective, acceptable and practical health care: A meta-analysis. *PloS one*, *5*(10), e13196. https://doi.org/10.1371/journal.pone.0013196

Baer, R. A. (2003). Mindfulness Training as a Clinical Intervention: A Conceptual and Empirical Review. *Clinical psychology: Science and practice*, *10*(2), 125–143. https://doi.org/10.1093/clipsy/bpg015

Bailer, J., Rist, F., Müller, T., Mier, D., Diener, C., Ofer, J.,. . . Witthöft, M. (2013). Erfassung von Krankheitsangst mit dem Short Health Anxiety Inventory (SHAI). *Verhaltenstherapie & Verhaltensmedizin*, *34*(4), 378–398.

Bandelow, B. (2016). *PAS - Panik- und Agoraphobie-Skala: Manual* (2., aktualisierte Auflage). Göttingen: Hogrefe.

Barak, A., Hen, L., Boniel-Nissim, M., & Shapira, N.'a. (2008). A Comprehensive Review and a Meta-Analysis of the Effectiveness of Internet-Based Psychotherapeutic Interventions. *Journal of Technology in Human Services*, *26*(2-4), 109–160. https://doi.org/10.1080/15228830802094429

Barlow, D. H., Farchione, T. J., Bullis, J. R., Gallagher, M. W., Murray-Latin, H., Sauer-Zavala, S.,. . . Cassiello-Robbins, C. (2017). The Unified Protocol for Transdiagnostic Treatment of Emotional Disorders Compared With Diagnosis-Specific Protocols for Anxiety Disorders: A Randomized Clinical Trial. *JAMA psychiatry.* Advance online publication. https://doi.org/10.1001/jamapsychiatry.2017.2164

Barlow, D. H., Farchione, T. J., Sauer-Zavala, S., Latin, H. M., Ellard, K. K., Bullis, J. R.,. . . Cassiello-Robbins, C. (2018). *Unified Protocol for Transdiagnostic Treatment of Emotional Disorders: Therapist Guide* (Second Edition). New York: Oxford University Press.

Bleichhardt, G., & Hiller, W. (2007). Hypochondriasis and health anxiety in the German population. *British journal of health psychology*, *12*(Pt 4), 511–523. https://doi.org/10.1348/135910706X146034

Boettcher, J., Aström, V., Påhlsson, D., Schenström, O., Andersson, G., & Carlbring, P. (2014). Internet-based mindfulness treatment for anxiety disorders: A randomized controlled trial. *Behavior Therapy*, *45*(2), 241–253. https://doi.org/10.1016/j.beth.2013.11.003

Boswell, J. F., Anderson, L. M., & Barlow, D. H. (2014). An idiographic analysis of change processes in the unified transdiagnostic treatment of depression. *Journal of consulting and clinical psychology*, *82*(6), 1060–1071. https://doi.org/10.1037/a0037403

Bullinger, M., & Kirchberger, I. (2011). *SF-36, Fragebogen zum Gesundheitszustand* (2., erg. u. überarb. Aufl.). Göttingen: Hogrefe.

Cuijpers, P., Marks, I. M., van Straten, A., Cavanagh, K., Gega, L., & Andersson, G. (2009). Computer-aided psychotherapy for anxiety disorders: A meta-analytic review. *Cognitive behaviour therapy*, *38*(2), 66–82. https://doi.org/10.1080/16506070802694776

European Medicines Agency Committee For Medicinal Products For Human Use (CHMP). Guideline on Non-inferiority Margin. Retrieved from http://www.ema.europa.eu/docs/en_GB/document_library/Scientific_guideline/2009/09/WC500003636.pdf

Farchione, T. J., Fairholme, C. P., Ellard, K. K., Boisseau, C. L., Thompson-Hollands, J., Carl, J. R.,. . . Barlow, D. H. (2012). Unified Protocol for Transdiagnostic Treatment of Emotional Disorders: A Randomized Controlled Trial. *Behavior Therapy*, *43*(3), 666–678. https://doi.org/10.1016/j.beth.2012.01.001

Gallagher, M. W., Sauer-Zavala, S. E., Boswell, J. F., Carl, J. R., Bullis, J., Farchione, T. J., & Barlow, D. H. (2013). The Impact of the Unified Protocol for Emotional Disorders on Quality of Life. *International Journal of Cognitive Therapy*, *6*(1). https://doi.org/10.1521/ijct.2013.6.1.57

Grossman, P., Niemann, L., Schmidt, S., & Walach, H. (2004). Mindfulness-based stress reduction and health benefits. *Journal of psychosomatic research*, *57*(1), 35–43. https://doi.org/10.1016/S0022-3999(03)00573-7

Harvey, A. G. (2004). *Cognitive behavioural processes across psychological disorders: A transdiagnostic approach to research and treatment*: Oxford University Press, USA.

Hedman, E., Andersson, G., Andersson, E., Ljótsson, B., Rück, C., Asmundson, G. J. G., & Lindefors, N. (2011). Internet-based cognitive-behavioural therapy for severe health anxiety: randomised controlled trial. *The British journal of psychiatry : the journal of mental science*, *198*(3), 230–236. https://doi.org/10.1192/bjp.bp.110.086843

Hedman, E., Axelsson, E., Andersson, E., Lekander, M., & Ljótsson, B. (2016). Exposure-based cognitive-behavioural therapy via the internet and as bibliotherapy for somatic symptom disorder and illness anxiety disorder: Randomised controlled trial. *The British journal of psychiatry : the journal of mental science*, *209*(5), 407–413. https://doi.org/10.1192/bjp.bp.116.181396

Heimberg, R. G., Horner, K. J., Juster, H. R., Safren, S. A., Brown, E. J., Schreiner, F. R., & Liebowitz, M. R. (1999). Psychometric properties of the Liebowitz Social Anxiety Scale. *Psychological medicine*, *29*, 199–212. Retrieved from https://www.cambridge.org/core/services/aop-cambridge-core/content/view/6891D37D00A9BEC179E61C8BFF30F08A/S0033291798007879a.pdf/psychometric-properties-of-the-liebowitz-social-anxiety-scale.pdf

Hofmann, S. G., Sawyer, A. T., Witt, A. A., & Oh, D. (2010). The effect of mindfulness-based therapy on anxiety and depression: A meta-analytic review. *Journal of consulting and clinical psychology*, *78*(2), 169–183. https://doi.org/10.1037/a0018555

Jacobi, F., Höfler, M., Siegert, J., Mack, S., Gerschler, A., Scholl, L.,. . . Wittchen, H.-U. (2014). Twelve-month prevalence, comorbidity and correlates of mental disorders in Germany: The Mental Health Module of the German Health Interview and Examination Survey for Adults (DEGS1-MH). *International journal of methods in psychiatric research*, *23*(3), 304–319. https://doi.org/10.1002/mpr.1439

Kabat-Zinn, J. (1982). An outpatient program in behavioral medicine for chronic pain patients based on the practice of mindfulness meditation: Theoretical considerations and preliminary results. *General hospital psychiatry*, *4*(1), 33–47.

Kroenke, K., & Spitzer, R. L. (2002). The PHQ-9: a new depression diagnostic and severity measure. *Psychiatric annals*, *32*(9), 509–515.

Lovas, D. A., & Barsky, A. J. (2010). Mindfulness-based cognitive therapy for hypochondriasis, or severe health anxiety: A pilot study. *Journal of anxiety disorders*, *24*(8), 931–935. https://doi.org/10.1016/j.janxdis.2010.06.019

Löwe, B., Decker, O., Müller, S., Brähler, E., Schellberg, D., Herzog, W., & Herzberg, P. Y. (2008). Validation and standardization of the Generalized Anxiety Disorder Screener (GAD-7) in the general population. *Medical care*, *46*(3), 266–274. https://doi.org/10.1097/MLR.0b013e318160d093

Löwe, B., Kroenke, K., Herzog, W., & Gräfe, K. (2004). Measuring depression outcome with a brief self-report instrument: Sensitivity to change of the Patient Health Questionnaire (PHQ-9). *Journal of affective disorders*, *81*(1), 61–66. https://doi.org/10.1016/S0165-0327(03)00198-8

Luberto, C. M., Magidson, J. F., & Blashill, A. J. (2017). A Case Study of Individually Delivered Mindfulness-Based Cognitive Behavioral Therapy for Severe Health Anxiety. *Cognitive and behavioral practice*, *24*(4), 484–495. https://doi.org/10.1016/j.cbpra.2016.10.001

Mansell, W., Harvey, A., Watkins, E., & Shafran, R. (2009). Conceptual Foundations of the Transdiagnostic Approach to CBT. *Journal of Cognitive Psychotherapy*, *23*(1), 6–19. https://doi.org/10.1891/0889-8391.23.1.6

McManus, F., Surawy, C., Muse, K., Vazquez-Montes, M., & Williams, J. M. G. (2012). A randomized clinical trial of mindfulness-based cognitive therapy versus unrestricted services for health anxiety (hypochondriasis). *Journal of consulting and clinical psychology*, *80*(5), 817–828. https://doi.org/10.1037/a0028782

Newby, J. M., Mahoney, A. E.J., Mason, E. C., Smith, J., Uppal, S., & Andrews, G. (2016). Pilot trial of a therapist-supported internet-delivered cognitive behavioural therapy program for health anxiety. *Internet Interventions*, *6*, 71–79. https://doi.org/10.1016/j.invent.2016.09.007

Newby, J. M., Twomey, C., Yuan Li, S. S., & Andrews, G. (2016). Transdiagnostic computerised cognitive behavioural therapy for depression and anxiety: A systematic review and meta-analysis. *Journal of affective disorders*, *199*, 30–41. https://doi.org/10.1016/j.jad.2016.03.018

Richards, D., & Richardson, T. (2012). Computer-based psychological treatments for depression: A systematic review and meta-analysis. *Clinical psychology review*, *32*(4), 329–342. https://doi.org/10.1016/j.cpr.2012.02.004

Rosenbaum, P. R., & Rubin, D. B. (1985). Constructing a Control Group Using Multivariate Matched Sampling Methods That Incorporate the Propensity Score. *The American Statistician*, *39*(1), 33–38.

Rozental, A., Kottorp, A., Boettcher, J., Andersson, G., & Carlbring, P. (2016). Negative Effects of Psychological Treatments: An Exploratory Factor Analysis of the Negative Effects Questionnaire for Monitoring and Reporting Adverse and Unwanted Events. *PloS one*, *11*(6), e0157503. https://doi.org/10.1371/journal.pone.0157503

Rozental, A., Magnusson, K., Boettcher, J., Andersson, G., & Carlbring, P. (2017). For better or worse: An individual patient data meta-analysis of deterioration among participants receiving Internet-based cognitive behavior therapy. *Journal of consulting and clinical psychology*, *85*(2), 160–177. https://doi.org/10.1037/ccp0000158

Salkovskis, P. M., Rimes, K. A., Warwick, H. M. C., & Clarke, D. M. (2002). The Health Anxiety Inventory: Development and validation of scales for the measurement of health anxiety and hypochondriasis. *Psychological Medicine*, *32*(05). https://doi.org/10.1017/S0033291702005822

Saxon, D., Ashley, K., Bishop-Edwards, L., Connell, J., Harrison, P., Ohlsen, S.,. . . Barkham, M. (2017). A pragmatic randomised controlled trial assessing the non-inferiority of counselling for depression versus cognitive-behaviour therapy for patients in primary care meeting a diagnosis of moderate or severe depression (PRaCTICED): Study protocol for a randomised controlled trial. *Trials*, *18*(1), 93. https://doi.org/10.1186/s13063-017-1834-6

Spijkerman, M. P. J., Pots, W. T. M., & Bohlmeijer, E. T. (2016). Effectiveness of online mindfulness-based interventions in improving mental health: A review and meta-analysis of randomised controlled trials. *Clinical psychology review*, *45*, 102–114. https://doi.org/10.1016/j.cpr.2016.03.009

Stangier, U., & Heidenreich, T. (2005). Die Liebowitz Soziala Angst-Skala (LSAS). In Collegium Internationale Psychiatriae Scalarum (Ed.), *Internationale Skalen für Psychiatrie.* Göttingen: Beltz.

Tyrer, P., Salkovskis, P., Tyrer, H., Wang, D., Crawford, M. J., Dupont, S.,. . . Barrett, B. (2017). Cognitive-behaviour therapy for health anxiety in medical patients (CHAMP): A randomised controlled trial with outcomes to 5 years. *Health technology assessment (Winchester, England)*, *21*(50), 1–58. https://doi.org/10.3310/hta21500

Ware Jr, J. E., Kosinski, M., & Keller, S. D. (1996). A 12-Item Short-Form Health Survey: Construction of scales and preliminary tests of reliability and validity. *Medical care*, *34*(3), 220–233.

Williams, M. J., McManus, F., Muse, K., & Williams, J. M. G. (2011). Mindfulness-based cognitive therapy for severe health anxiety (hypochondriasis): An interpretative phenomenological analysis of patients' experiences. *The British journal of clinical psychology*, *50*(4), 379–397. https://doi.org/10.1111/j.2044-8260.2010.02000.x

Wittchen, H.-U., Wunderlich, U., Gruschwitz, S., & Zaudig, M. (1997). *SKID I. Strukturiertes Klinisches Interview für DSM-IV. Achse I*. Göttingen: Hogrefe.

Zagorscak, P., Heinrich, M., Sommer, D., Wagner, B., & Knaevelsrud, C. (2018). Benefits of Individualized Feedback in Internet-Based Interventions for Depression: A Randomized Controlled Trial. *Psychotherapy and psychosomatics*, *87*(1), 32–45. https://doi.org/10.1159/000481515

*- English Translation –*

**Request for Approval from the Ethics Committee of Freie Universität Berlin on the conduct of a scientific study**

Title: Feasibility, acceptance, and effectiveness of the online intervention iUP.

Names and addresses of the participating scientists

Dr. Johanna Böttcher, Freie Universität Berlin, Department of Education and Psychology, Clinical Psychology and Psychotherapy Unit,
Habelschwerdter Allee 45, 14195 Berlin, Tel.: 030-838-56569, E-Mail: [johanna.boettcher@fu-berlin.de](mailto:b.renneberg@fu-berlin.de)

Dipl. -Psych. Carmen Schäuffele, Free University of Berlin, Department of Education and Psychology, Clinical-Psychological Intervention Unit,

Habelschwerdter Allee 45, 14195 Berlin, Tel.: 030-838-63696 E-mail: carmen.schaeuffele@fu-berlin.de

## Summary of the subject of the application

The aim of the planned studies is to investigate the feasibility, acceptance, and efficacy of the online-based intervention "iUP". iUP is a 10-week online-based therapy program for people with anxiety, depressive and somatic symptom disorders. In this program, participants learn new content on a weekly basis and work on their management of emotions through exercises. There will be a guided and unguided version. In the guided version, participants will receive weekly personalized feedback on their work in the program from a personal advisor. In the unguided version, participants will receive automated, standardized feedback. Three studies are planned: In a first study ("pilot study"), the guided version of the iUP will be evaluated in a sample of individuals with anxiety disorders (social anxiety disorder, generalized anxiety disorder, panic disorder, agoraphobia), depressive, and somatic disorder (somatic symptom disorder and illness anxiety disorder). A second study will evaluate the effectiveness of the guided version for people with primary depressive symptoms and compare it to another active treatment. In the third study, the effectiveness of the unguided iUP for people with illness anxiety will be examined in more detail and compared with an unguided mindfulness program.

## Aim of the studies

The so-called emotional disorders, which include anxiety, depressive, and somatic symptom disorders, have a high prevalence in the general population and high comorbidity rates (Jacobi et al., 2014). Comorbidity rates are not only burdensome for those with the disorders, but also present challenges for treatment. Traditional cognitive behavior therapy manuals focus only on one disorder and usually do not provide guidance on how to manage comorbid symptoms. To address multiple mental disorders simultaneously in therapy, so-called transdiagnostic therapy approaches have been developed (Harvey, 2004; Mansell, Harvey, Watkins, & Shafran, 2009). The Unified Protocol is a transdiagnostic treatment program that addresses all emotional disorders (Barlow et al., 2018). The Unified Protocol has been evaluated face-to-face in several studies in the United States, and evidence of its effectiveness is found, particularly for anxiety disorders (Barlow et al., 2017; Farchione et al., 2012). Its effectiveness for depressive disorders has only been examined in single-case studies (Boswell, Anderson, & Barlow, 2014). Findings on the effectiveness of the Unified Protocol are also lacking for other emotional disorders such as somatic symptom disorder and illness anxiety disorder.

Adapting the Unified Protocol in an online context would allow comorbid emotional disorders to be treated simultaneously, taking advantage of the benefits of online therapy. Numerous studies and meta-analyses have demonstrated the effectiveness of online-based therapy in the areas of depression, anxiety disorders, and health anxiety (Andersson & Cuijpers, 2009; Andrews, Cuijpers, Craske, McEvoy, & Titov, 2010; Barak, Hen, Boniel-Nissim, & Shapira, 2008; Cuijpers et al., 2009; Hedman et al, 2011; Hedman, Axelsson, Andersson, Lekander, & Ljótsson, 2016; Newby, Mahoney et al, 2016). Online interventions provide sufferers with easy, accessible, flexible, and relatively anonymous access to evidence-based therapy (Andersson & Titov, 2014).

In the planned studies, an Internet-based version of the Unified Protocol (iUP) will be evaluated. In order to make iUP accessible in the German language area, the English therapy manual will be translated and tested on patients with one or more emotional disorders in three studies for feasibility, acceptance, and effectiveness.

**Study 1:** The pilot study examines the feasibility, acceptability, and effectiveness of the guided version with a randomized controlled design in a sample of patients with anxiety, depressive, and somatic symptom disorders. Participants assigned to the wait-list control group will have access to the intervention after ten weeks.

**Study 2:** As mentioned, studies on the effectiveness of the UP in depression are lacking. Therefore, the aim of the second study is to investigate the transdiagnostic iUP in the guided version on a sample of depressed patients and to compare it with a depression-specific guided online intervention (TK-DepressionsCoach). The TK-DepressionsCoach is a guided six-week depression-specific online intervention developed at Freie Universität in cooperation with Techniker Krankenkasse. Similar to the planned guided iUP intervention, participants learn psychoeducational content and work on a homework assignment for one week until they receive personalized feedback. An initial study of the TK Depression Coach with N=1089 participants showed very large within-group effects for depressive symptomatology (d=1.20) (Zagorscak, Heinrich, Sommer, Wagner, & Knaevelsrud, 2018). A study on the TK-DepressionCoach is also currently ongoing with already more than 1000 participants. In Study 2, we would like to use the large sample of the TK-DepressionCoach to construct a control group for the iUP using matched sampling (z.B. Rosenbaum & Rubin, 1985). Matching of the two groups will be done using propensity score matching. Results to date indicate that the UP is as effective as disorder-specific therapy for anxiety disorders (Barlow et al., 2017) and that transdiagnostic online therapy achieves comparable effects to disorder-specific online therapy (Newby, Twomey, Yuan Li, & Andrews, 2016). Therefore, it is anticipated that transdiagnostic iUP will not be inferior to the disorder-specific online intervention TK-DepressionCoach.

**Study 3:** As mentioned, studies on the effectiveness of the Unified Protocol on individuals with health anxiety are pending, despite the fact that health anxiety is very common in the general population and is a rather underserved disorder (Bleichhardt & Hiller, 2007). Unguided interventions represent a starting point to further increase the availability of therapy. To date, no evidence of differences between a guided and unguided online intervention for health anxiety has been reported. A study by Hedman and colleagues (2016) demonstrated that unguided treatment programs are similarly effective as guided programs in treating illness anxiety. Therefore, the aim of the third study is to investigate the effectiveness of the unguided iUP for people with marked health anxiety. To this end, the iUP will be compared with an active control group completing unguided online mindfulness training using a randomized-controlled design. The promotion of mindfulness has become a major focus of cognitive behavioral therapy approaches in recent years. Meta-analyses confirm the effectiveness of mindfulness programs for a range of clinical and nonclinical samples (Baer, 2003; Grossman, Niemann, Schmidt, & Walach, 2004; . Hofmann, Sawyer, Witt, & Oh, 2010). In the area of health anxiety, several studies also support the effectiveness of mindfulness-based approaches (Lovas & Barsky, 2010; Luberto, Magidson, & Blashill, 2017; McManus, Surawy, Muse, Vazquez-Montes, & Williams, 2012). For anxiety disorders, there is preliminary evidence that Internet-based mindfulness promotion also leads to reductions in stress, anxiety, and depressive symptoms (Boettcher et al., 2014). Studies on the effects of Internet-based mindfulness programs for health anxiety have yet to be conducted.

## Methods and study design

### Recruitment and selection of participants

**Study 1-3**: Participants will be recruited via announcements in Internet health forums. Potential participants are informed on a study website about the aims, procedure, and possible benefits and risks of the planned study (see Appendix A, exemplary information letter for the pilot study). If participants are interested in taking part, they will be asked to complete and sign the consent form and send it via mail to the study management (see Appendix B, exemplary consent form for the pilot study). Participation in the study is voluntary. There is the option to interrupt or discontinue participation at any time without giving a reason. After the consent form has been received by the study management, participants are invited via e-mail to register with their e-mail address, a user name of their own choice, and a password.

**Study 1 and 2:** Participants will be included in two steps: In a first step, patients will complete demographic, cross-disorder, and disorder-specific questionnaires (see survey instruments). If participants are above the cut-off of at least one disorder-specific questionnaire at this point, they will be contacted via email to schedule a telephone interview.

In the telephone interview, the Structured Clinical Interview for DSM-IV is used to determine one or more diagnoses (Wittchen, Wunderlich, Gruschwitz, & Zaudig, 1997). The interviewers are the two applicants and honorary staff who are master's students in clinical psychology and who have been trained in conducting the SKID. All interviewers are bound by confidentiality.

**Study 3:** After informed consent, patients complete demographic, cross-disorder, and disorder-specific questionnaires (see survey instruments). If participants score above the cut-off on the illness anxiety subscale of the Short Health Anxiety Inventory, they will be included in the study. For this purpose, the cut-off is set at 20, a cut-off value that has been used in previous studies (Tyrer et al., 2017) and showed a good balance of specificity and sensitivity in the German validation study (Bailer et al., 2013).

Sample size

**Study 1:** Based on an expected large effect (Cohen's *d* = .8) in favor of the intervention group (one-sided t-test for independent samples, alpha=0.05) and a power of 80%, we target a sample of *N=42* participants. With a maximum expected drop-out of 15% to the post-survey, this results in a sample to be recruited of *N=60*.

**Study 2:** This study examined the non-inferiority of the iUP compared to a depression-specific online intervention. It is recommended that the noninferiority margin be set using historical results comparing the control intervention and placebo, often setting it at a size equal to 50% of the effect size observed in these studies (Althunian, Boer, Groenwold, & Klungel, 2017; European Medicines Agency Committee For Medicinal Products For Human Use [CHMP]). Meta-analyses show a pooled effect size of *d=*.78 for supervised online interventions for depressive disorders (Richards & Richardson, 2012). Accordingly, the non-inferiority margin Δ is set at 0.39 (.78x0.5). Translated to the primary outcome measure PHQ-9, this margin means that the lower bound of the 95% confidence interval around the mean difference between treatments must not exceed -2.7 PHQ points (assumed SD of difference value = 6.9 (Saxon et al., 2017)). To show this, with a power of 95% and alpha=0.05, a sample of *N=143 is* required. With a maximum expected drop-out of 15% to the post-survey, this results in a sample to be recruited of *N=168*.

**Study 3**: Based on a small to medium expected effect (Cohen's *d* =. 4) in favor of the iUP program (one-sided t-test for independent samples, alpha = 0.05) and a power of 80%, we target a sample of *N=156* participants. With a maximum expected drop-out of 15% to the post-survey, this results in a sample to be recruited of *N=184*.

Inclusion and **exclusion criteria**

Participants will be included if they **a)** are at least 18 years of age, **b)** have access to the Internet, **c)** are able to participate in a telephone interview, **d)** for **Study 1:** have a primary SKID diagnosis of Generalized Anxiety Disorder, Social Anxiety Disorder, Panic Disorder, Agoraphobia, Depression (single episode or recurrent), Dysthymia, Somatic Symptom Disorder with predominant illness anxiety or an illness anxiety disorder, for **Study 2**: have a primary diagnosis of Depression (single episode or recurrent) or Dysthymia established with the SKID, and for **Study 3**: have elevated illness anxiety, defined as a score above 20 on the illness anxiety subscale of the Short Health Anxiety Inventory , **e)** are not already receiving other psychotherapeutic treatment or counseling, **f)** have been on a stable dose of medication for three months, should they be taking medication for anxiety or depression, **g)** do not have acute psychotic symptoms or substance dependence, and **h)** are not acutely suicidal.

Suicidal ideation is assessed with the suicide item of the PHQ-9 (Kroenke & Spitzer, 2002; Löwe, Kroenke, Herzog, & Gräfe, 2004). If participants answer >1 to this item during the screening process, their suicide risk is evaluated during the diagnostic interview. Interviews with potentially suicidal participants will not be conducted by master's students but by one of the two applicants. The first applicant is a licensed CBT therapist and the second applicant is in advanced CBT training. Participants with acute suicide risk will be excluded from the study and referred to local psychiatrists or psychotherapists.

### Reimbursement

Participants in all three studies will not be compensated for their participation in the online-based intervention.

### Study design

**Study 1:** The planned study follows a randomized controlled design. Participants will be randomly assigned to the intervention group or the waiting group. Randomization will be based on random number sequence implemented in the online platform, independent of the investigator. After the telephone interview, participants in the intervention group will receive access to the online-based intervention via email. Participants in the waiting group will receive access to the iUP after a ten-week waiting period.

**Study 2:** The study is a non-randomized, non-inferiority trial. Participants in the iUP will be matched to an existing sample of N>2000 participants of the TK-DepressionCoach to construct a control group. A propensity score - the estimated probability of being in the group depending on previously defined possible covariates - will be determined for all participants. Covariates on which the propensity score is to be estimated include severity of depression, comorbid disorders, prior psychotherapeutic treatment, medication use, age, gender, schooling, and relationship status. This procedure allows to determine exactly one "nearest neighbor" from the TK-DepressionsCoach sample for each participant of the iUP group based on the propensity score and in this way to create a control group as comparable and equal in size as possible. The change in depressive symptomatology will then be compared between the disorder-specific TK-DepressionCoach and the transdiagnostic iUP.

**Study 3:** The study will follow a randomized-controlled design with an active control group. The active control group will complete an unaccompanied mindfulness program over the course of 10 weeks, with mindfulness exercises based on Mindfulness-Based Stress Reduction (Kabat-Zinn, 1982). Participants in the mindfulness group will receive a weekly introduction and then practice a mindful attitude independently using provided audio files. Participants will be encouraged to log their experience and progress within the program.

### Intervention - iUP

The use of the online-based intervention is designed for 10 weeks. The modules deal with motivation and goals (module 1), emotions (module 2), mindfulness (module 3), cognitive flexibility (modules 4 and 5), emotion avoidance (module 6), tolerance of physical sensations (module 7), confrontation with situations or thoughts that trigger strong emotions and that the participants have previously avoided (modules 8 and 9), and relapse prevention (module 10). In each module, participants find psychoeducational content and exercises, which they record in record sheets. We recommend that participants plan at least 60 minutes per week to work on the content, but participants can decide for themselves how much time to invest and how to divide up the work. In the guided version of the iUP, regular contact with the advisors is foreseen. After working on a module for a week, participants receive personalized feedback on their work and access to the next module. In the unguided version of the iUP, participants receive automated feedback and are then given access to the next module. In both versions, participants are informed at the beginning of the intervention that they can contact us via message if they have any questions or difficulties.

**Online advisors:** The online consultants are the applicant Carmen Schäuffele and students of clinical psychology who are writing their master's thesis in the project. Both applicants have completed training in the Unified Protocol at Boston University. Master's students receive an introduction to the program and then practice writing online feedback on sample patients. For the first treatment case, the drafted feedback is reviewed by one of the two applicants and corrected as necessary. Supervision sessions with the first applicant will take place weekly over the period of the study.

### Survey instruments

Clinical Interview: A telephone interview using the SKID (Wittchen et al., 1997) will be conducted at two time points in Studies 1 and 2: Pre-treatment for diagnosis and post-treatment for reassessment of diagnoses made.

Participants will complete a short questionnaire at the beginning of each new module and a more comprehensive battery of measurements before, halfway through, and after treatment. A follow-up survey will be administered at 3, 6, and 12 months. In addition, after some modules, participants will complete process measures on skills to be learned in that module

The PHQ-4 patient health questionnaire (Kroenke, Spitzer, Williams, & Löwe, 2009; Löwe et al., 2010) will be used for a weekly measure of change. For Study 1 and 2, which will include depressed individuals, an item asking about suicidality will also be used to provide clues about possible suicidality. Participants who report increased suicidal thoughts in one of the weekly surveys (suicide item>1) will be contacted via telephone by one of the applicants.

The primary measure used in Study 1 is the General Health Questionnaire GHQ-12 (Goldberg et al.; Schmitz, Kruse, & Tress, 1999), in Study 2 the Patient Health Questionnaire PHQ-9 (Kroenke & Spitzer, 2002; Löwe et al., 2004), and in Study 3 the short form of the Health Anxiety Inventory (Bailer et al., 2013; Salkovskis, Rimes, Warwick, & Clarke, 2002).

Other diagnosis-specific questionnaires include: the Patient Health Questionnaire PHQ-9 for depression (Kroenke & Spitzer, 2002; Löwe et al., 2004), the Liebowitz Social Anxiety Scale for social anxiety disorder (Heimberg et al., 1999; Stangier & Heidenreich, 2005), the short form of the Health Anxiety Inventory for somatic symptom disorder and illness anxiety disorder (Bailer et al., 2013; Salkovskis et al., 2002), the Panic and Agoraphobia Scale for panic disorder and agoraphobia (Bandelow, 2016), the Generalized Anxiety Disorder Screener GAD-9 for generalized anxiety disorder (Löwe et al., 2008), and the Short-Form Health Survey SF-12 for health-related quality of life (Bullinger & Kirchberger, 2011; Ware Jr, Kosinski, & Keller, 1996). In addition, impairments related to somatic symptoms are assessed with a visual analog scale.

Process measures include the Southhampton Mindfulness Questionnaire SMQ for mindfulness (Chadwick et al.., 2008), the Emotion Regulation Questionnaire ERQ reassessment subscale for cognitive flexibility (Gross & John, 2003; Abler & Kessler, 2009), the Brief Experiential Avoidance Scale BEAQ for emotion avoidance (Gámez et al.., 2014), and the Behavioral Activation Depression Scale for behavioral-level avoidance (Fuhr et al., 2016; Manos, Kanter, & Luo, 2011).

To assess negative effects of treatments, the Negative Effects Questionnaire NEQ is completed after treatment (Rozental, Kottorp, Boettcher, Andersson, & Carlbring, 2016).

## Risks and possible consequences for the participants

No blood or tissue samples will be taken in the planned studies. There will be no testing of a drug. There is no deception of study participants.

Participation in the iUP intervention is associated with prospective benefits. Studies suggest that internet-based interventions, both guided and unguided, may be helpful in reducing anxious and depressive symptoms (see Introduction). For the Unified Protocol, there is also evidence for face-to-face treatment leading to reductions in anxiety symptoms (Barlow et al., 2017; Farchione et al., 2012) and increased quality of life (Gallagher et al., 2013). However, randomized controlled trials are pending for depressive and somatic symptom disorders.

Based on empirical findings to date, the potential risk of participating in iUP can be considered low. Approximately 5% of participants in similar online therapy programs report experiencing an increase in stress, anxiety, or other unpleasant feelings. In the vast majority of cases, these are temporary and have no negative consequences (Rozental, Magnusson, Boettcher, Andersson, & Carlbring, 2017).

It is reasonable to assume, based on the results of previous studies, that participation in an unaccompanied mindfulness intervention (active control group study 3) also leads to an improvement in general well-being and a reduction in anxiety and depressive symptomatology (Boettcher et al., 2014; Spijkerman, Pots, & Bohlmeijer, 2016). Previous findings also show that mindfulness practice does not lead to a worsening of health anxiety despite focusing attention on physical processes (McManus et al., 2012; Williams, McManus, Muse, & Williams, 2011).

Participants are informed in detail about the expected benefits and potential risks (see Appendix A).

1. Dealing with incidental findings

For Studies 1 and 2, participants will be included in the study based on the screening questionnaires and clinical diagnostic interview, and for Study 3, based on a score above the cut-off on the Short Health Anxiety Inventory.

Due to the inclusion of depressed patients, instruments containing items on suicidality are also used in the study. Patients will be educated on how to deal with potential suicidal crises as part of the study and will be provided with contact points and phone numbers. If participants indicate that they are suffering from suicidal thoughts in the screening or the surveys within the intervention, they will be contacted via telephone by the investigators.

The results of the SKID interview are not communicated to the patient as a diagnosis due to the prohibition of remote diagnosis. Such a remote diagnosis would not be supported by a somatic clarification and would not fulfill the legal requirements of personal face-to-face clarification.

1. Data protection precautions

The collection, storage, and evaluation of study data is subject to the provisions of the Berlin Data Protection Act. The following measures are taken at the different study stages to ensure the security of the data.

**6.1. Survey**

The collected data are initially pseudonymized. Personal data will only be collected to the extent necessary to conduct the study. Prior to registration, participants will be asked to print out and sign the consent form (see Appendix B) and send it via mail to the study management. The consent form will include name, address, phone number, and email address. The consent forms will be kept in a locked cabinet for ten years.

Online, general demographic information (age, gender, marital status, schooling, current and previous psychotherapeutic treatment, and medication use) is collected, as well as pseudonym/username and password for registration. An e-mail address is also requested. This is used for the purpose of password resets, reminders to log in regularly, and contact at follow-up times. The profile data is exclusively requested and stored within a password-protected online platform (using SSL-secured data transmission) and deleted after the last collection.

Participants can request the deletion of their data for as long as the data set can be assigned to their person. Following the analysis, the data is archived in anonymized form and used exclusively for scientific purposes.

**6.2 Storage**

The screening data of the first questionnaire survey is collected on the same platform as the online program and stored in compliance with legal requirements and in compliance with established security standards.

The data generated within the scope of the program ("usage data", such as frequency of logins) is stored pseudonymously. The security of the Internet server is continuously monitored by a security scan. No further information of the participants (e.g. movement profiles, access to Internet pages) is stored. The data is sent via SSL encryption to a server of the German server provider Hetzner (Hetzner Online GmbH), whose servers are certified according to ISO/IEC 27001 and thus meet high European data protection standards.

After completion of the studies, the fully anonymized data are made publicly available via the Internet database Open Science Framework. This procedure serves to ensure good scientific work.

**6.3 Evaluation**

The retrieval of the stored pseudonymized data for research/evaluation purposes is password protected. The password is only accessible to the project investigators of the research project under review. All project staff members agree in writing to maintain confidentiality about personal data and to comply with the Berlin Data Protection Act.

No evaluations or reports are produced that allow conclusions to be drawn about individual persons. Small groups of people (e.g. age groups) are not shown separately.

## Study funding

The study is financed from budget funds. There is no request from a third-party funder for an ethical review.

## Explanation

I am aware of the Regulations for the Ethical Review of Research Projects of the Department of Education and Psychology of Freie Universität Berlin. I am aware that according to §19 of the Berlin Data Protection Act (BlnDSG), I am obliged to create a file and procedure description for automated processing of personal and personal-related data and to make this available to the official data protection officer of the FU Berlin according to §19a.

An application for ethical review of the research project "Feasibility, Acceptability, and Effectiveness of the Online Intervention iUP" has not yet been submitted to any other site.

Johanna Böttcher Carmen Schäuffele

Berlin, the March 2018

**Appendix A:** Exemplary participant information for the participants of the pilot study

| **Information sheet dated 14.03.2018 about the study  "iUP - an online-based program for people with anxiety and depression "** | |
| --- | --- |
| Place of the study | Frei Universität Berlin  Department of Clinical Psychology and Psychotherapy  Habelschwerdter Allee 45, D-14195 Berlin |
|  |  |
| Responsible | Dr. Johanna Böttcher (FU Berlin) johanna.boettcher@fu-berlin.de |

**Dear interested party,**

we are pleased that you are considering trying the online program "iUP" as part of our study. We are investigating iUP as part of a scientific study conducted by the Freie Universität Berlin. In the following, we would like to inform you about the aims and the procedure of the study as well as about data protection.

**Background**

We all know fears, worry, or are sometimes depressed. This is completely normal! However, if these fears, worries, or dejection are very pronounced and a strong symptom distress arises, psychologists speak of a mental disorder. Because these disorders focus on intense feelings such as fear or sadness, they are also called "emotional disorders." The emotional disorders include, for example, the anxiety disorders or depressive disorders.

The online program iUP, which we would like to investigate in this study, starts exactly there. The iUP is based on the Unified Protocol treatment concept and was originally developed at Boston University. The special feature of the program is that all emotional disorders, i.e. all disorders in which emotions play a role, can be treated with it - even if someone has more than one such emotional disorder.

**Targets**

The aim of this study is to test the feasibility and effectiveness of iUP for people with emotional disorders. Initial research in the USA has already shown that the Unified Protocol can help people with such disorders. This study will now examine whether the Unified Protocol is also effective when used as an online program, i.e., when all therapy is delivered over the Internet (see below).

In order to test the effect of iUP, the users of the program (treatment group) must be compared with a group of people who do not use the program during the same period and who do not use any other treatment for their psychological complaints (waiting control group). These individuals are on a waiting list, so to speak - after the end of the treatment group's usage period (10 weeks), the waiting control group is given the opportunity to use the program to the same extent as the treatment group before them. Comparing an active treatment group with a waiting control group is a necessary standard in the review of psychological programs.

By participating in our study, you would agree to be drawn into one of two user groups: either the treatment group, which begins active use of the program immediately, or the control group, which begins the program after a 10-week waiting period.

**Procedure**

Let us now explain the process of the study:

At the beginning we want to find out if the offered program is right for you. This clarification process is important because our approach is only suitable for people who suffer from an emotional disorder. The clarification process is as follows:

After you have agreed to participate in the study and sent us the consent form via mail, you can register. You will then complete a number of questionnaires online about behaviors and feelings. If the questionnaires indicate that you may have an emotional disorder, we will schedule a phone interview with you. In this telephone interview, we will ask you questions using a structured guideline to make sure that participation in the program is right for you. If the results of the phone interview also indicate that we can help you with your symptoms using iUP, you will be randomly assigned to the treatment group or the waiting control group. If you end up in the treatment group, we will immediately activate you for the program. An online counselor will be assigned to you. In the waiting control group, we will contact you again after 10 weeks and invite you to participate in the program.

Then you can start with the treatment. In total, the program is designed for 10 weeks, and you can decide for yourself how often or how intensively you want to complete the exercises. In the exercises, you learn skills for dealing with overwhelming feelings. This requires a lot of practice and prolonged engagement with the content. We therefore recommend that you work with the program for at least 60 minutes per week. Once a week, you will receive feedback from your personal counselor. We will also regularly ask you questions about your current complaints in order to be able to follow short-term developments. Your progress will be automatically stored in pseudonymized form.

In order for us to evaluate whether the program is working, we will ask you to complete a short questionnaire weekly and the questionnaires from the beginning halfway through and again after the program. In addition, we will contact you again via e-mail 3, 6 and 12 months after the end of the program and ask you to fill in the questionnaires again.

After program completion, we will also ask you to participate in another telephone interview. Here we would like to check how your complaints have developed.

**Voluntariness and anonymity**

Participation in the study is voluntary. You can terminate your participation in this study at any time and without giving reasons, without incurring any disadvantages.

The data and personal communications collected in the course of this study will be treated confidentially. For example, all project staff who have personal data at their disposal through direct contact with you are subject to the duty of confidentiality. Furthermore, the results of the study will be published in anonymized form, i.e. without your data being able to be assigned to your person.

**Privacy**

We collect various personal data from you as part of the study. Firstly, we collect your name, address, telephone number, and e-mail address with the consent form. Then we collect the data from the telephone interview as well as the data from the questionnaires that you fill out on the computer at different times during the study and following the completion of the study. In addition, your personally chosen username, password, and an email address will be stored. Finally, we collect data on the use of the program, which are stored automatically, e.g. how often you log into the program.

We have taken the following measures to ensure the confidentiality of your data:

1) The postal consent form is kept separately from the other data in a locked cabinet.

2) The transmission of your data via the Internet is exclusively encrypted. Thereby

a technology is used that is also used, for example, by banks in the internet-based

use payment transactions (SSL encryption).

3) All digital data is stored pseudonymously without mentioning your name. After the last data collection, the data is then anonymized. This means that it is no longer possible for anyone to associate the collected data with your name. Until the last data collection, you can request the deletion of all data collected from you. Once the record is anonymized, we can no longer identify your record. Therefore, we can only honor your request to delete your data up to the last collection.

3) The program is hosted on an external server of the German server provider Hetzner (Hetzner Online GmbH), which is certified according to ISO/IEC 27011 and thus meets the highest security standards.

4) All data and personal communications collected as part of this study are subject to

the duty of confidentiality.

The fully anonymized data are made publicly available via the Internet database Open Science Framework. This procedure serves to ensure good scientific work. Other researchers can thus, for example, reproduce the evaluation or test an alternative evaluation.

**Benefits and risks of participating in the study**

Participation in the study is associated with a probable benefit, which is offset by a certain cost and possible risks.

*Potential benefits:* Participation in the iUP program is associated with likely benefits. Studies suggest that internet-based treatment programs may be helpful in reducing anxious and depressive symptoms. With iUP, you can practice skills for managing feelings that are known to be linked to increases in mental health and well-being. Because the online-based program implemented in this study has not yet been investigated, the expected positive effects are hypotheses that may be supported by scientific results after the study is completed.

*Potential risks:* Similar to drug treatments, psychological programs can have side effects. Approximately 5% of participants in similar programs report experiencing an increase in stress, anxiety, or other unpleasant feelings. In the vast majority of cases, these are temporary and have no negative consequences. Nevertheless, we encourage you to contact us or your online counselor directly if you feel a worsening of your symptoms. We would also like to point out that the use of the online program cannot replace psychological, psychiatric, or medical treatment.

Participation in the study and use of iUP is free of charge and will not be compensated.

**Ethics Committee**

The study was approved by the Ethics Committee of the Department of Education and Psychology at Freie Universität Berlin.

**Appendix B:** Exemplary Postal Consent Form for the Pilot Study.

| **Informed consent for the study  "iUP - an online-based treatment for people with anxiety and depression ".** | |
| --- | --- |
| Place of the study | Frei Universität Berlin  Department of Clinical Psychology and Psychotherapy  Habelschwerdter Allee 45, 14195 Berlin |
|  |  |
| Responsible | Dr. Johanna Böttcher (FU Berlin) johanna.boettcher@fu-berlin.de |

| Name |  |
| --- | --- |
| Address |  |
| Phone number |  |
| E-mail address |  |

Please read the following points carefully!

Please ask if there is anything you do not understand or would like to know!

- - - 1. I have carefully read and understood the pilot study information available on the study website. My questions related to participation in this study have been answered satisfactorily. I will receive a copy of my signed informed consent form.
      2. I understand that the data and personal communications collected as part of this study are subject to confidentiality. I agree that persons involved in the study may inspect my original data, but under strict observance of confidentiality. Confidentiality will be strictly maintained throughout the study. The data will initially be stored pseudonymously and anonymized after the last survey. I consent to the data processing of my personal data in accordance with the participant information I received.
      3. I am participating in the study voluntarily. I can revoke my consent to participate at any time and without giving reasons and request the deletion of my data up to the last survey without incurring any disadvantages as a result.

I confirm by my signature that I am willing to participate in the study.

| Place, date | Your signature |
| --- | --- |
| Place, date | Signature of person involved in the study (please leave blank). |

Please send the completed and signed consent form to

**Dr. Johanna Böttcher, Freie Universität Berlin, Department of Clinical Psychology and Psychotherapy, Habelschwerdter Allee 45, 14195 Berlin**

A copy of the copy signed by us will be sent to you via mail.

Literature

Althunian, T. A., Boer, A. de, Groenwold, R. H. H., & Klungel, O. H. (2017). Defining the noninferiority margin and analysing noninferiority: An overview. *British journal of clinical pharmacology*, *83*(8), 1636-1642. https://doi.org/10.1111/bcp.13280

Andersson, G., & Cuijpers, P. (2009). Internet-based and other computerized psychological treatments for adult depression: A meta-analysis. *Cognitive behaviour therapy*, *38*(4), 196-205. https://doi.org/10.1080/16506070903318960

Andersson, G., & Titov, N. (2014). Advantages and limitations of Internet-based interventions for common mental disorders. *World psychiatry : official journal of the World Psychiatric Association (WPA)*, *13*(1), 4-11. https://doi.org/10.1002/wps.20083

Andrews, G., Cuijpers, P., Craske, M. G., McEvoy, P., & Titov, N. (2010). Computer therapy for the anxiety and depressive disorders is effective, acceptable and practical health care: A meta-analysis. *PloS one*, *5*(10), e13196. https://doi.org/10.1371/journal.pone.0013196

Baer, R. A. (2003). Mindfulness Training as a Clinical Intervention: A Conceptual and Empirical Review. *Clinical psychology: Science and practice*, *10*(2), 125-143. https://doi.org/10.1093/clipsy/bpg015

Bailer, J., Rist, F., Müller, T., Mier, D., Diener, C., Ofer, J.,. . . Witthöft, M. (2013). Assessment of illness anxiety with the Short Health Anxiety Inventory (SHAI). *Behavior Therapy & Behavioral Medicine, 34*(4*),* 378-398.

Bandelow, B. (2016). *PAS - Panic and agoraphobia scale: Manual* (2nd, updated edition). Göttingen: Hogrefe.

Barak, A., Hen, L., Boniel-Nissim, M., & Shapira, N.'a. (2008). A Comprehensive Review and a Meta-Analysis of the Effectiveness of Internet-Based Psychotherapeutic Interventions. *Journal of Technology in Human Services*, *26*(2-4), 109-160. https://doi.org/10.1080/15228830802094429

Barlow, D. H., Farchione, T. J., Bullis, J. R., Gallagher, M. W., Murray-Latin, H., Sauer-Zavala, S.,. . . Cassiello-Robbins, C. (2017). The Unified Protocol for Transdiagnostic Treatment of Emotional Disorders Compared With Diagnosis-Specific Protocols for Anxiety Disorders: A Randomized Clinical Trial. *JAMA psychiatry.* Advance online publication. https://doi.org/10.1001/jamapsychiatry.2017.2164

Barlow, D. H., Farchione, T. J., Sauer-Zavala, S., Latin, H. M., Ellard, K. K., Bullis, J. R.,. . . Cassiello-Robbins, C. (2018). *Unified Protocol for Transdiagnostic Treatment of Emotional Disorders: Therapist Guide* (Second Edition). New York: Oxford University Press.

Bleichhardt, G., & Hiller, W. (2007). Hypochondriasis and health anxiety in the German population. *British journal of health psychology*, *12*(Pt 4), 511-523. https://doi.org/10.1348/135910706X146034

Boettcher, J., Aström, V., Påhlsson, D., Schenström, O., Andersson, G., & Carlbring, P. (2014). Internet-based mindfulness treatment for anxiety disorders: A randomized controlled trial. *Behavior Therapy*, *45*(2), 241-253. https://doi.org/10.1016/j.beth.2013.11.003

Boswell, J. F., Anderson, L. M., & Barlow, D. H. (2014). An idiographic analysis of change processes in the unified transdiagnostic treatment of depression. *Journal of consulting and clinical psychology*, *82*(6), 1060-1071. https://doi.org/10.1037/a0037403

Bullinger, M., & Kirchberger, I. (2011). *SF-36, health status questionnaire* (2nd, supplemented and revised ed.). Göttingen: Hogrefe.

Cuijpers, P., Marks, I. M., van Straten, A., Cavanagh, K., Gega, L., & Andersson, G. (2009). Computer-aided psychotherapy for anxiety disorders: A meta-analytic review. *Cognitive behaviour therapy*, *38*(2), 66-82. https://doi.org/10.1080/16506070802694776

European Medicines Agency Committee For Medicinal Products For Human Use (CHMP). Guideline on Non-inferiority Margin. Retrieved from http://www.ema.europa.eu/docs/en_GB/document_library/Scientific_guideline/2009/09/WC500003636.pdf

Farchione, T. J., Fairholme, C. P., Ellard, K. K., Boisseau, C. L., Thompson-Hollands, J., Carl, J. R.,. . . Barlow, D. H. (2012). Unified Protocol for Transdiagnostic Treatment of Emotional Disorders: A Randomized Controlled Trial. *Behavior Therapy*, *43*(3), 666-678. https://doi.org/10.1016/j.beth.2012.01.001

Gallagher, M. W., Sauer-Zavala, S. E., Boswell, J. F., Carl, J. R., Bullis, J., Farchione, T. J., & Barlow, D. H. (2013). The Impact of the Unified Protocol for Emotional Disorders on Quality of Life. *International Journal of Cognitive Therapy*, *6*(1). https://doi.org/10.1521/ijct.2013.6.1.57

Grossman, P., Niemann, L., Schmidt, S., & Walach, H. (2004). Mindfulness-based stress reduction and health benefits. *Journal of psychosomatic research*, *57*(1), 35-43. https://doi.org/10.1016/S0022-3999(03)00573-7

Harvey, A. G. (2004). *Cognitive behavioural processes across psychological disorders: A transdiagnostic approach to research and treatment*: Oxford University Press, USA.

Hedman, E., Andersson, G., Andersson, E., Ljótsson, B., Rück, C., Asmundson, G. J. G., & Lindefors, N. (2011). Internet-based cognitive-behavioural therapy for severe health anxiety: randomised controlled trial. *The British journal of psychiatry : the journal of mental science*, *198*(3), 230-236. https://doi.org/10.1192/bjp.bp.110.086843

Hedman, E., Axelsson, E., Andersson, E., Lekander, M., & Ljótsson, B. (2016). Exposure-based cognitive-behavioural therapy via the internet and as bibliotherapy for somatic symptom disorder and illness anxiety disorder: Randomised controlled trial. *The British journal of psychiatry : the journal of mental science*, *209*(5), 407-413. https://doi.org/10.1192/bjp.bp.116.181396

Heimberg, R. G., Horner, K. J., Juster, H. R., Safren, S. A., Brown, E. J., Schreiner, F. R., & Liebowitz, M. R. (1999). Psychometric properties of the Liebowitz Social Anxiety Scale. *Psychological medicine*, *29*, 199-212. Retrieved from https://www.cambridge.org/core/services/aop-cambridge-core/content/view/6891D37D00A9BEC179E61C8BFF30F08A/S0033291798007879a.pdf/psychometric-properties-of-the-liebowitz-social-anxiety-scale.pdf

Hofmann, S. G., Sawyer, A. T., Witt, A. A., & Oh, D. (2010). The effect of mindfulness-based therapy on anxiety and depression: A meta-analytic review. *Journal of consulting and clinical psychology*, *78*(2), 169-183. https://doi.org/10.1037/a0018555

Jacobi, F., Höfler, M., Siegert, J., Mack, S., Gerschler, A., Scholl, L.,. . . Wittchen, H.-U. (2014). Twelve-month prevalence, comorbidity and correlates of mental disorders in Germany: The Mental Health Module of the German Health Interview and Examination Survey for Adults (DEGS1-MH). *International journal of methods in psychiatric research*, *23*(3), 304-319. https://doi.org/10.1002/mpr.1439

Kabat-Zinn, J. (1982). An outpatient program in behavioral medicine for chronic pain patients based on the practice of mindfulness meditation: Theoretical considerations and preliminary results. *General hospital psychiatry*, *4*(1), 33-47.

Kroenke, K., & Spitzer, R. L. (2002). The PHQ-9: a new depression diagnostic and severity measure. *Psychiatric annals*, *32*(9), 509-515.

Lovas, D. A., & Barsky, A. J. (2010). Mindfulness-based cognitive therapy for hypochondriasis, or severe health anxiety: A pilot study. *Journal of anxiety disorders*, *24*(8), 931-935. https://doi.org/10.1016/j.janxdis.2010.06.019

Löwe, B., Decker, O., Müller, S., Brähler, E., Schellberg, D., Herzog, W., & Herzberg, P. Y. (2008). Validation and standardization of the Generalized Anxiety Disorder Screener (GAD-7) in the general population. *Medical care*, *46*(3), 266-274. https://doi.org/10.1097/MLR.0b013e318160d093

Löwe, B., Kroenke, K., Herzog, W., & Gräfe, K. (2004). Measuring depression outcome with a brief self-report instrument: Sensitivity to change of the Patient Health Questionnaire (PHQ-9). *Journal of affective disorders*, *81*(1), 61-66. https://doi.org/10.1016/S0165-0327(03)00198-8

Luberto, C. M., Magidson, J. F., & Blashill, A. J. (2017). A Case Study of Individually Delivered Mindfulness-Based Cognitive Behavioral Therapy for Severe Health Anxiety. *Cognitive and behavioral practice*, *24*(4), 484-495. https://doi.org/10.1016/j.cbpra.2016.10.001

Mansell, W., Harvey, A., Watkins, E., & Shafran, R. (2009). Conceptual Foundations of the Transdiagnostic Approach to CBT. *Journal of Cognitive Psychotherapy*, *23*(1), 6-19. https://doi.org/10.1891/0889-8391.23.1.6

McManus, F., Surawy, C., Muse, K., Vazquez-Montes, M., & Williams, J. M. G. (2012). A randomized clinical trial of mindfulness-based cognitive therapy versus unrestricted services for health anxiety (hypochondriasis). *Journal of consulting and clinical psychology*, *80*(5), 817-828. https://doi.org/10.1037/a0028782

Newby, J. M., Mahoney, A. E.J., Mason, E. C., Smith, J., Uppal, S., & Andrews, G. (2016). Pilot trial of a therapist-supported internet-delivered cognitive behavioural therapy program for health anxiety. *Internet Interventions*, *6*, 71-79. https://doi.org/10.1016/j.invent.2016.09.007

Newby, J. M., Twomey, C., Yuan Li, S. S., & Andrews, G. (2016). Transdiagnostic computerised cognitive behavioural therapy for depression and anxiety: A systematic review and meta-analysis. *Journal of affective disorders*, *199*, 30-41. https://doi.org/10.1016/j.jad.2016.03.018

Richards, D., & Richardson, T. (2012). Computer-based psychological treatments for depression: A systematic review and meta-analysis. *Clinical psychology review*, *32*(4), 329-342. https://doi.org/10.1016/j.cpr.2012.02.004

Rosenbaum, P. R., & Rubin, D. B. (1985). Constructing a Control Group Using Multivariate Matched Sampling Methods That Incorporate the Propensity Score. *The American Statistician*, *39*(1), 33-38.

Rozental, A., Kottorp, A., Boettcher, J., Andersson, G., & Carlbring, P. (2016). Negative Effects of Psychological Treatments: An Exploratory Factor Analysis of the Negative Effects Questionnaire for Monitoring and Reporting Adverse and Unwanted Events. *PloS one*, *11*(6), e0157503. https://doi.org/10.1371/journal.pone.0157503

Rozental, A., Magnusson, K., Boettcher, J., Andersson, G., & Carlbring, P. (2017). For better or worse: An individual patient data meta-analysis of deterioration among participants receiving Internet-based cognitive behavior therapy. *Journal of consulting and clinical psychology*, *85*(2), 160-177. https://doi.org/10.1037/ccp0000158

Salkovskis, P. M., Rimes, K. A., Warwick, H. M. C., & Clarke, D. M. (2002). The Health Anxiety Inventory: Development and validation of scales for the measurement of health anxiety and hypochondriasis. *Psychological Medicine*, *32*(05). https://doi.org/10.1017/S0033291702005822

Saxon, D., Ashley, K., Bishop-Edwards, L., Connell, J., Harrison, P., Ohlsen, S.,. . . Barkham, M. (2017). A pragmatic randomised controlled trial assessing the non-inferiority of counselling for depression versus cognitive-behaviour therapy for patients in primary care meeting a diagnosis of moderate or severe depression (PRaCTICED): Study protocol for a randomised controlled trial. *Trials*, *18*(1), 93. https://doi.org/10.1186/s13063-017-1834-6

Spijkerman, M. P. J., Pots, W. T. M., & Bohlmeijer, E. T. (2016). Effectiveness of online mindfulness-based interventions in improving mental health: A review and meta-analysis of randomised controlled trials. *Clinical psychology review*, *45*, 102-114. https://doi.org/10.1016/j.cpr.2016.03.009

Stangier, U., & Heidenreich, T. (2005). The Liebowitz social anxiety scale (LSAS). In Collegium Internationale Psychiatriae Scalarum (Ed.), *International scales of psychiatry.* Göttingen: Beltz.

Tyrer, P., Salkovskis, P., Tyrer, H., Wang, D., Crawford, M. J., Dupont, S.,. . . Barrett, B. (2017). Cognitive-behaviour therapy for health anxiety in medical patients (CHAMP): A randomised controlled trial with outcomes to 5 years. *Health technology assessment (Winchester, England)*, *21*(50), 1-58. https://doi.org/10.3310/hta21500

Ware Jr, J. E., Kosinski, M., & Keller, S. D. (1996). A 12-Item Short-Form Health Survey: Construction of scales and preliminary tests of reliability and validity. *Medical care*, *34*(3), 220-233.

Williams, M. J., McManus, F., Muse, K., & Williams, J. M. G. (2011). Mindfulness-based cognitive therapy for severe health anxiety (hypochondriasis): An interpretative phenomenological analysis of patients' experiences. *The British journal of clinical psychology*, *50*(4), 379-397. https://doi.org/10.1111/j.2044-8260.2010.02000.x

Wittchen, H.-U., Wunderlich, U., Gruschwitz, S., & Zaudig, M. (1997). *SKID I. Structured clinical interview for DSM-IV. Axis I*. Göttingen: Hogrefe.

Zagorscak, P., Heinrich, M., Sommer, D., Wagner, B., & Knaevelsrud, C. (2018). Benefits of Individualized Feedback in Internet-Based Interventions for Depression: A Randomized Controlled Trial. *Psychotherapy and psychosomatics*, *87*(1), 32-45. https://doi.org/10.1159/000481515
